# Interpretable self-supervised transformers for resting-state EEG analysis in Alzheimer's disease

**DOI:** 10.3389/fninf.2026.1899573

**Published:** 2026-08-27

**Authors:** Syeda Shamaila Zareen, Nada Alzaben, Usman Ahmad, Talha Mahboob Alam, Asaad Algarni, Junsong Wang

**Affiliations:** 1 College of Applied Sciences, Shenzhen University, Shenzhen, China; 2 Guangdong Key Laboratory for Biomedical Measurements and Ultrasound Imaging, National-Regional Key Technology Engineering Laboratory for Medical Ultrasound, School of Biomedical Engineering, Shenzhen University Medical School, Shenzhen, China; 3 School of Artificial Intelligence, Shenzhen Technology University, Shenzhen, China; 4 Department of Computer Sciences, College of Computer and Information Sciences, Princess Nourah Bint Abdulrahman University, Riyadh, Saudi Arabia; 5 Department of AI & Data Science, FAST School of Computing, National University of Computer and Emerging Sciences, Islamabad, Pakistan; 6 Department of Computer Science, Norwegian University of Science and Technology, Trondheim, Norway; 7 Department of Computer Science, Faculty of Computing and Information Technology, King Abdulaziz University, Rabigh, Saudi Arabia

**Keywords:** Alzheimer's disease, deep learning, interpretable attention, resting-state EEG, self-supervised learning, spatio-temporal transformer, transfer learning

## Abstract

Existing EEG-based methods have been constrained by limited availability of labeled data, hand-crafted features, poor spatio-temporal modeling, sub-optimal cross-hardware performance, and lack of interpretability due to being expensive and intrusive. To address these limitations, this study introduces innovative neural signal decoding techniques for cognitive state modeling in order to enhance the potential of AI-integrated models for early detection of Alzheimer's disease. This research introduces a self-supervised spatio-temporal transformer (STT-EEG) for early detection of Alzheimer's disease from resting-state EEG. This framework includes four key components: first, self-supervised pretraining on 111 healthy controls using masked auto-encoding and temporal order prediction to learn robust generalisable representations. Second, a novel spatial attention module (SAM) that explicitly captures both long-range temporal dependencies and channel interactions, reflecting the distributed network pathology of AD; third, cross-dataset transfer learning from 64-channel BioSemi to 19-channel Nihon Kohden systems, which showed strong hardware generalization; and finally, analyses of attention rollout and channel perturbation for clinically interpretable insights. The model was trained in a subject-wise 5-fold cross-validation fashion on the SRM dataset and fine-tuned on the OpenNeuro dataset (ds004504) consisting of 36 AD and 29 CN. On the same dataset, the accuracy of STT-EEG was 96.42% for AD vs. CN classification. The most significant improvement +7.08% was made with the help of self-supervised pretraining, followed by data augmentation +6.30% and the SAM +4.86%, as was confirmed in the ablation studies. For continuous prediction of MMSE scores, the Pearson correlation of the model was 0.872 and the mean absolute error (MAE) was 2.34 points. Regions of T3–T6, P3–Pz–P4 and O1–O2 were identified as areas of attention-based interpretability, which were consistent with the known neuropathology of AD that involved the temporoparietal lobe. STT-EEG strengths include the high interpretability of the framework, its spatio-temporal attention, and the fact that STT-EEG is a self-supervised learning method and can be used in an efficient and generalizable way.

## 1 Introduction

The World Health Organization (WHO) estimates that currently over 55 million people suffer from dementia, and that will increase substantially in the next decades. Alzheimer's disease (AD) is a progressive and irreversible neurodegenerative disease representing the most common type of dementia in the world with about 70% of cases worldwide (Jeong, 2004). AD prevalence increases significantly with age, particularly in individuals over 65. Given its high economic and social costs, global health authorities have prioritized dementia on the public health agenda, emphasizing the need for increased awareness, early diagnosis, and improved care and support for affected individuals. It is important to make an early diagnosis of AD for a number of reasons: (1) early diagnosis allows patients and families to be alerted and to plan accordingly; (2) drug treatments for psychiatric symptoms are most likely to be effective at an early stage; (3) effective treatment of symptoms can reduce health care costs and the societal burden; and (4) effective therapeutic prevention interventions can be developed, which may improve therapeutic outcomes. Diagnostic methods involve extensive neuropsychological assessments, exclusion of other causes and several biomarkers such as the Mini-Mental State Examination (MMSE) (Folstein et al., 1975), Montreal cognitive assessment (MoCA), blood markers, CSF markers and several neuroimaging techniques (Li et al., 2026).

Extensive work has been done to investigate the pathophysiology of AD using cutting-edge neuroimaging technology. Gray matter atrophy in the medial temporal lobe and hippocampus as well as the temporoparietal regions is identified in structural magnetic resonance imaging (MRI) (Babiloni et al., 2009; Moretti et al., 2007). In vivo visualization of amyloid-β deposition and tau neurofibrillary tangle accumulation is possible using positron emission tomography (PET); CT is used to obtain complementary structural information. However, these techniques also have a number of practical challenges: they are not suitable for early detection of pathological changes, are expensive, require expertise for interpretation, and are not widely available in clinical practice, especially in resource-poor settings (Ahamed et al., 2025).

EEG is an alternative method which is low cost, readily available, highly efficient and also has excellent temporal resolution (Miltiadous et al., 2023b,a). EEG is directly related with functional changes in the cerebral cortex and could be used to quantify the neuronal degeneration related to the progression of AD, even before clear behavioral symptoms can be detected (Tzimourta et al., 2019). EEG signals offer various analysis perspectives: temporal dynamics, frequency domain structure and source imaging (Pascual-Marqui et al., 1995).

Three characteristic changes of the EEG in AD patients in comparison to healthy controls have been established: (1) diffuse slowing, with a decrease of spectral power in higher frequencies (alpha, beta, gamma) and an increase in lower frequencies (delta, theta) (Wang et al., 2015); (2) reduction in complexity, as reflected in entropy-based measures (Sun et al., 2020; Zúñiga et al., 2024); and (3) reduction in functional connectivity between cortical areas, denoting a disruption of large-scale brain networks (Dauwels et al., 2010; Vinck et al., 2011). These neurophysiological changes are thought to represent cholinergic dysfunction, cortical hyperexcitability, and neocortical disconnection (Newman et al., 2012).

Recently, machine learning (ML) based approaches have been demonstrated to have potential in AD detection, such as the method of eLORETA source localization combined with ML classifiers which achieved 76.6% accuracy in predicting 1-year cognitive decline in ADMCI patients (Babiloni et al., 2026) and multiscale functional connectivity analysis using majority voting with 91.97% accuracy in MCI detection (Deng et al., 2024). For MRI data, the accuracy of 99.45% for a four-class AD classification was obtained by modifying EfficientNetV2B3 by Ahamed et al. (2025). Furthermore, research using EEG has investigated methods of feature extraction such as entropy analysis (Tzimourta et al., 2019), spectral and complexity biomarkers (Al-Nuaimi et al., 2021), automated machine learning pipelines (Chedid et al., 2022) and EEG microstates as markers of diseases (Miltiadous et al., 2023a). To increase the data quality and the data reproducibility from different data sets, there have been standardized pre-processing frameworks like PREP (Bigdely-Shamlo et al., 2015) and Autoreject (Jas et al., 2017). Yet several key areas of research are still not addressed:

- Most studies have small cohorts of subjects with only one dataset and do not make use of the large volumes of unlabeled EEG data found in publicly available datasets, thus reducing the potential for deep-learning models to learn generalizable representations and to risk overfitting (Ghasemzadeh et al., 2024).
- Traditional methods rely on manually designed features that are obtained through signal processing pipelines, such as spectral power, entropy, and connectivity, but these methods do not capture long-range temporal dependencies in resting-state EEG (Zheng et al., 2023; Hjorth, 1970).
- Few evaluations have considered how well models work for various recording devices, channel montages, and clinical procedures, making it challenging to translate the models into clinical practice (Miltiadous et al., 2023b; Wang and Lei, 2023).
- The models used in deep learning are hard to interpret and most studies are focused on AD vs CN classification instead of predicting the cognitive decline (Ahamed et al., 2025; Babiloni et al., 2026).

In order to overcome these research gaps, we propose a Self-Supervised Spatio-Temporal Transformer (STT-EEG) to predict Alzheimer's disease (AD) at an early stage based on resting-state EEG. The research proposed five major innovations:

1. Pretraining with masked autoencoding on healthy controls in the SRM dataset (Hatlestad-Hall et al., 2022) to learn general EEG representations, minimizing overfitting and improving generalization when fine-tuned to limited labeled AD data.
2. A novel Spatial Attention Module (SAM) extending the standard transformers to explicitly model both temporal and cross-channel spatial interactions, which captures the distributed network-level pathology of AD.
3. Attention rollout and channel perturbation analysis create spatio-temporal importance maps to highlight which EEG channels and time points are important to make predictions in a transparent and clinically trustworthy manner.
4. Simultaneous binary classification (AD vs. CN) and MMSE regression, offering both diagnostic (disease presence) and prognostic (cognitive decline severity) information from a shared representation.

## 2 Literature review

Electroencephalography (EEG) has emerged as a promising, cost-effective, and non-invasive tool for early Alzheimer's disease (AD) detection (Jeong, 2004). The EEG alterations of AD patients are characterized by diffuse slowing of power, low power in alpha, beta, and gamma bands, and high power in delta and theta bands, low signal complexity, and disturbed functional connectivity (Lejko et al., 2020). Some of the most common biomarkers derived from these neurophysiological signals have been extensively used in traditional machine learning approaches, such as entropy-based features and support vector machines (Tzimourta et al., 2019) and spectral and complexity features and random forest classifiers (Al-Nuaimi et al., 2021) or even an automated machine learning pipeline that fused statistical, spectral, and connectivity features with ensemble classifiers Chedid et al. (2022). Recently, (Zheng et al., 2023) used a combination of spectral power, complexity and synchronization features with random forest classification and achieved a 95.86% accuracy on the OpenNeuro ds004504 dataset, while Miltiadous et al. (2023b) developed an EEG benchmark for AD and frontotemporal dementia (FTD) research. Despite these advances, traditional feature based methods are fundamentally limited: they are dependent on hand crafted features which cannot capture complex spatio-temporal dynamics, tend to over-fit on small data sets, and are not suited to generality across different recording hardware and clinical protocols. Furthermore, most of these studies have classified binary AD and CN, and not predicted continuous cognitive decline, limiting the clinical utility of these works (Ghasemzadeh et al., 2024; Miltiadous et al., 2023b).

To overcome these limitation, the deep learning architectures have been increasingly used for the identification of AD through EEG. EEGNet (Lawhern et al., 2018), DeepConvNet (Schirrmeister et al., 2017) and EEGInception (Zhang et al., 2021) are such CNN models that can perform end-to-end feature learning from raw EEG signals. The use of transformer-based architecture has taken the field one step further by including long-range temporal dependency using self-attention. EEGConformer (Song et al., 2022) integrates CNN-based feature extraction with transformer-based temporal-spatial attention mechanism, and PatchTST (Nie et al., 2023) presents patch-based time-series modeling. To explicitly model spatial interactions between EEG channels, recent state-of-the-art models like LUNA (Chen et al., 2025) and Trace (Ma et al., 2026) use learned-query cross-attention or temporal routing.

Foundation models pre-trained on a large-scale unlabeled EEG corpora have been introduced as a powerful paradigm to tackle the lack of labeled pathological EEG data. LaBraM (Jiang et al., 2024) uses masked autoencoding on large amounts of EEG data, such as TUH EEG and Sleep EDF, to obtain the best results in transfer learning for different EEG benchmarks. BIOT (Yang et al., 2024) adopts contrastive learning to represent biosignals, and CBraMod (Wang et al., 2024) proposes a criss-cross attention model that is pre-trained on around 2,500 hours of diverse brain signals, and CSBrain (Zhou et al., 2025) introduces a cross-scale spatiotemporal foundation model for EEG decoding. Self-supervised learning methods like masked autoencoding and temporal order prediction have demonstrated efficacy in capturing robust EEG representations without requiring labels (Jiang et al., 2024; Mohsenvand et al., 2019) and EEG-specific data augmentation techniques (Zhang et al., 2017; Lashgari et al., 2020; Thulasidasan et al., 2019) have also been shown to improve generalization of models. In light of these developments, current methods are still limited by the following reasons: limited labeled data, lack of spatio-temporal modeling, sub-optimal performance across different types of hardware, and lack of interpretability; hence the motivation for self-supervised spatio-temporal transformers with explicit spatial attention and attention-based explainability.

This paper is organized as follows: In Section 2, a brief literature review is discussed. In Section 3, the description of datasets, the preprocessing pipeline, and the full model are detailed. In Section 4, experimental details are provided. In Section 5 results are presented, which comprise classification accuracy, ablation studies, attention-based interpretability analysis, and comparisons with baseline methods. Section 6 discusses the clinical implications and limitations. Section 7 presents the conclusion of the overall work and directions for future studies.

## 3 Materials and methods

### 3.1 Datasets and preprocessing

**Figure 1:**
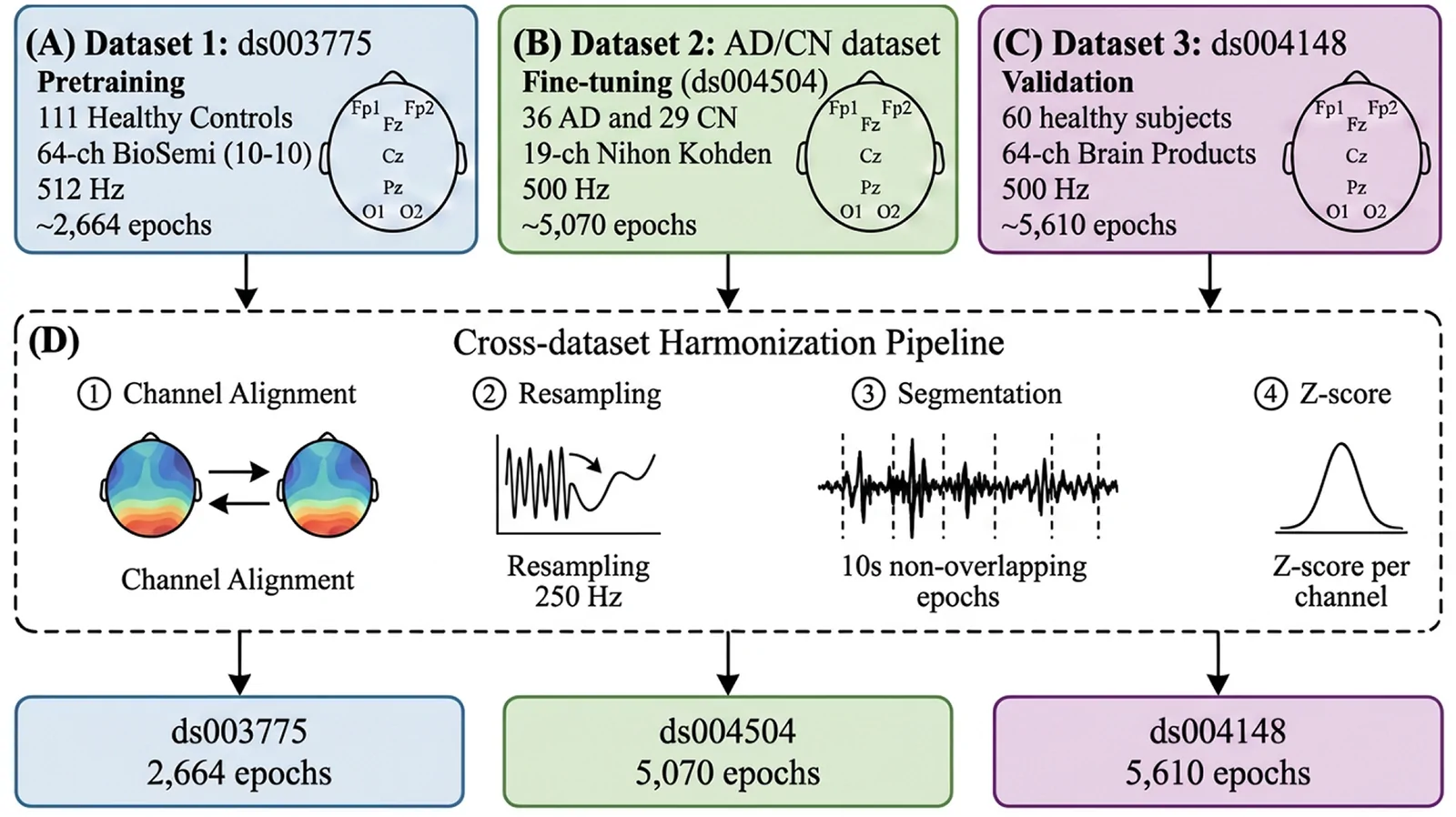
Schematic overview of the three EEG datasets and the cross-dataset harmonization pipeline. **(A)** SRM dataset (ds003775): Using 111 healthy controls with a 64 channel BioSemi system for self-supervised pretraining. **(B)** AD/CN dataset (ds004504): 36 Alzheimer's disease patients and 29 cognitively normal controls with 19-channel Nihon Kohden system. **(C)** Resting state and cognitive task data (ds004148) 60 healthy subjects with BrainProducts system of 64 channels. **(D)** Cross-dataset harmonization pipeline: channel alignment, resampling at 250 Hz, segmenting into 10 s non over-lapping epochs and z scoring per channel. The AD/CN dataset was used for fine-tuning and evaluation.

In this study, three resting-state electroencephalography (EEG) datasets were used that are publicly available. All datasets are made available under CC0 licenses and in the format of the Brain Imaging Data Structure (BIDS). The first dataset (OpenNeuro ds004504) was used for downstream Alzheimer's disease (AD) classification, the second dataset (OpenNeuro ds003775) was used to pre-train the model in a large-scale self-supervised manner, and the third dataset (OpenNeuro ds004148) was used as an independent validation set for the pre-trained model. To avoid information leakage, all the preprocessing tasks (e.g., normalization and augmentation) were completed separately in each training fold. Figure 1 shows an overview of the three EEG datasets and the cross-dataset harmonization pipeline.

#### 3.1.1 Alzheimer's disease EEG dataset (OpenNeuro ds004504)

The main downstream data were resting-state eyes-closed EEG data collected from 65 subjects: 36 Alzheimer's disease (AD) and 29 cognitively normal (CN) healthy controls (Miltiadous et al., 2023b). EEG was acquired using a clinical acquisition system of Nihon Kohden EEG-2100 at the 2nd Department of Neurology at AHEPA General Hospital, Thessaloniki, Greece. 19 electrodes, following the international 10–20 system (Fp1, Fp2, F7, F3, Fz, F4, F8, T3, C3, Cz, C4, T4, T5, P3, Pz, P4, T6, O1, O2), with A1 and A2 mastoid electrodes used for impedance monitoring and referencing were used to record signals. The recordings were done with 10 μV/mm sensitivity and 500 Hz sampling rate and the participants were seated with their eyes closed throughout the recording. The average duration of the recordings was around 13 min. The Mini-Mental State Examination (MMSE) (Folstein et al., 1975) was used to evaluate cognitive status. The AD group had a mean MMSE score of 17.75 ± 4.5 while the CN group had a mean score of 30.0 ± 0.0. Preprocessed EEG data were used. The preprocessing pipeline used included: (1) Butterworth band-pass filtering between 0.5–45 Hz, (2) re-referencing to A1-A2, (3) removal of high amplitude artifacts with Artifact Subspace Reconstruction (ASR), and (4) removal of ocular and muscular artifacts with ICA using ICLabel, which automatically rejects high amplitude artifacts.

#### 3.1.2 SRM resting-state EEG dataset (OpenNeuro ds003775)

We used the SRM resting-state EEG dataset (Hatlestad-Hall et al., 2022) with 111 healthy participants resting-state eyes-closed EEG data from the University of Oslo, Norway to pre-train the self-supervised model. EEG signals were recorded from 64 electrodes using the extended 10-10 system, on a BioSemi ActiveTwo acquisition system. Four minutes of continuous resting-state EEG were recorded for each participant at 512 Hz. The accompanying metadata files included demographic information and neuropsychological assessment scores. The following preprocessing steps were automated: bad channel detection and interpolation, band pass filtering (0.5–45 Hz), average re-referencing and artifact rejection using ASR in the EEGLAB framework.

#### 3.1.3 Resting-State and cognitive task EEG dataset (OpenNeuro ds004148)

To provide an additional independent validation dataset, we used resting-state (eyes closed and open) and cognitive task (subtraction, music, memory) EEG data from OpenNeuro ds004148 (Wang and Lei, 2023) containing data from 60 healthy participants. EEG recordings were done with a 64-channel BrainProducts system (Brain Products GmbH, Germany) with a sampling frequency of 500 Hz. Impedances were maintained at < 5 kΩ. The raw EEG data was preprocessed using an automated pipeline. Bad channel detection and interpolation and rejection of epochs containing high-amplitude artifacts were performed as pre-processing steps.

#### 3.1.4 Demographic characteristics

The demographic characteristics of the AD and CN groups are shown in Table 1 for the purposes of our comparative analyses. The demographic analysis shows that the AD group matched the CN group in age, sex, and education level with no significant differences between groups (all *p*-values > 0.05). Groups were significantly different in MMSE scores (p < 0.001), as would be expected, confirming the status of AD as cognitively impaired. These demographic variables align with the demographics described in previous AD EEG studies (Miltiadous et al., 2023b).

**Table 1:** Demographic characteristics of the AD and CN study cohorts.

Characteristic	AD group (*n* = 36)	CN group (*n* = 29)	*p*-value
Age (years)	66.4 ± 7.9	67.9 ± 5.4	0.291^a^
Sex (male/female)	19/17	15/14	0.921^b^
Education (years)	10.25 ± 3.12	11.08 ± 2.87	0.276^a^
MMSE score	17.75 ± 4.50	30.0 ± 0.0	<0.001^a^*

Data are presented as mean ± standard deviation.

^a^Two-sample t-test; ^b^Chi-square test. *Statistical significance at α = 0.05 after Benjamini-Hochberg correction.

#### 3.1.5 Cross-dataset harmonization

One of the biggest difficulties in merging both datasets is that there are differences in the channel configurations and acquisition protocols. To achieve a common set of electrodes, the SRM dataset was restricted to the 19 electrodes common to both datasets: Fp1, Fp2, F7, F3, Fz, F4, F8, T3, C3, Cz, C4, T4, T5, P3, Pz, P4, T6, O1, and O2. The 10–10 and 10–20 systems were used to create channel correspondence, with each channel identified by a standard convention of electrode mapping. Both sets of data were subsequently resampled to a common frequency of 250 Hz so as to reduce computational complexity, while maintaining physiologically-relevant EEG rhythms within the δ (0.5–4 Hz), θ (4-8 Hz), α (8–13 Hz), β (13–30 Hz), and γ (30–45 Hz) bands. The continuous EEG recordings were divided into 10-second epochs (without overlaps) and resampled to *T* = 2500 temporal samples per epoch. Let the epochs be represented by matrices:


$$\begin{array}{l} \text{X}\in {\mathbb{R}}^{C\times T} \end{array}$$ (1)

where *C* = 19 is the number of channels. Epoch-wise z-score normalization was applied independently to each channel to reduce inter-subject variability and stabilize model training. For each channel *c*, the normalized signal is:


$$\begin{array}{l} {\tilde{x}}_{c}\left(t\right)=\frac{x_{c}\left(t\right)-\mu_{c}}{\sigma_{c}+\epsilon },\;\forall t\in \left[1,T\right] \end{array}$$ (2)

where $\mu_{c}=\frac{1}{T}\overset{T}{\underset{t=1}{\sum }}x_{c}\left(t\right)$, $\sigma_{c}=\sqrt{\frac{1}{T}\overset{T}{\underset{t=1}{\sum }}{\left(x_{c}\left(t\right)-\mu_{c}\right)}^{2}}$, and ϵ = 10^−8^ prevents division by zero.

The SRM dataset has much more EEG epochs than the AD/CN fine-tuning dataset, and it has been used for large-scale pretraining.

### 3.2 Self-supervised pretraining framework

**Figure 2:**
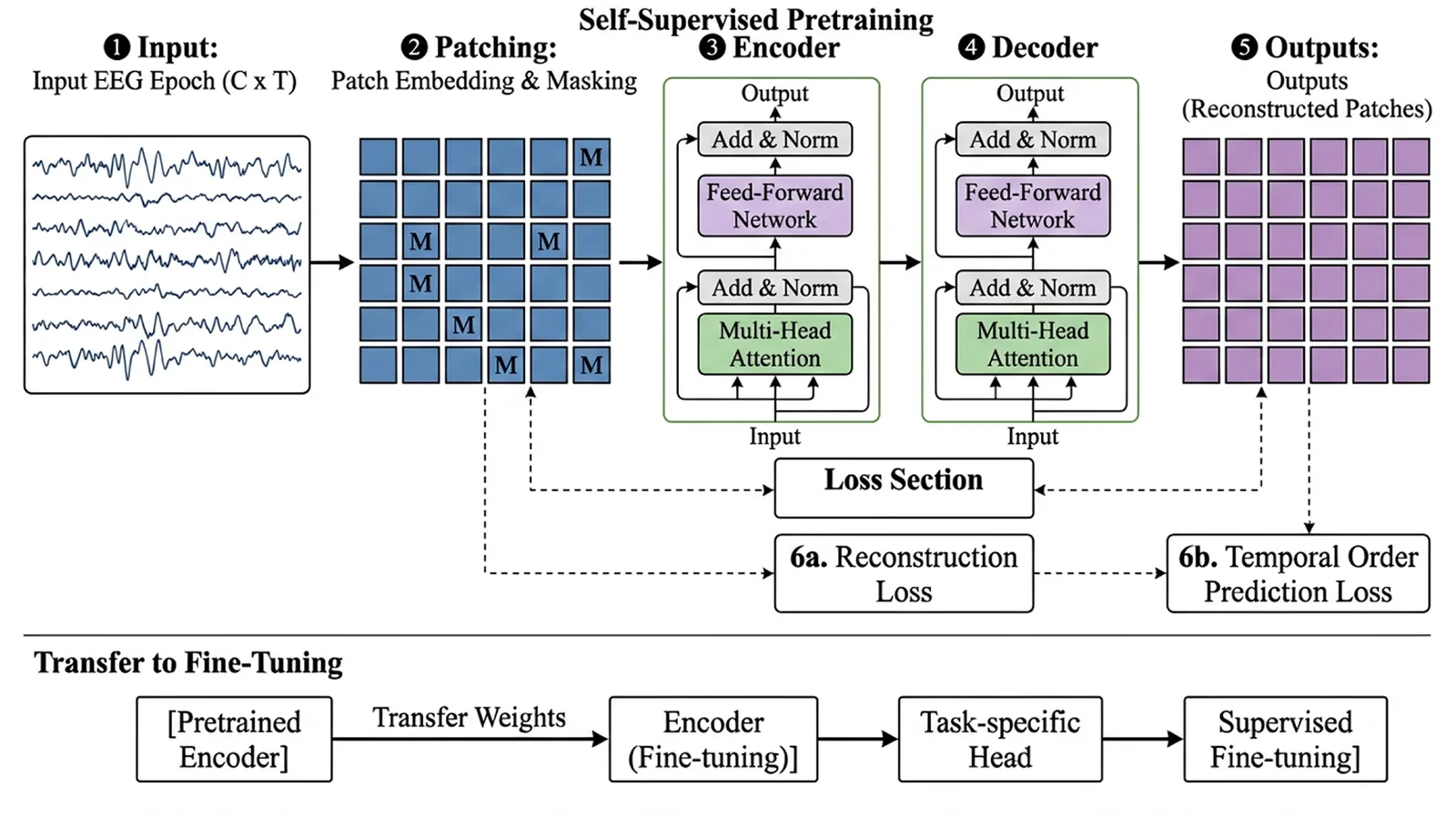
Self-supervised pretraining framework based on masked autoencoding and temporal order prediction. The EEG input **X** ∈ ℝ^*C*×*T*^ is converted into temporal patches using a 1D convolutional embedding layer. A hybrid masking strategy masks 75% of patches using both block and random masking. The Transformer encoder processes visible patches, while a lightweight decoder reconstructs masked patches using learnable mask tokens. The model is optimized with reconstruction loss and temporal order prediction loss, where ℒ_total_ = ℒ_recon_ + 0.1ℒ_temp_.

To address the limited availability of labeled Alzheimer's EEG data, we adopted a self-supervised learning (SSL) framework in which the transformer model was first pretrained on the large-scale unlabeled SRM dataset and subsequently fine-tuned on the AD classification dataset. The self-supervised pretraining framework combining masked autoencoding with temporal order prediction is illustrated in Figure 2.

#### 3.2.1 Masked EEG modeling and temporal prediction

The proposed SSL framework combines masked autoencoding and temporal order prediction to learn robust spatio-temporal EEG representations.

##### 3.2.1.1 Input representation

Each EEG epoch **X** ∈ ℝ^*C*×*T*^ was transformed into a sequence of temporal patches using a one-dimensional convolutional embedding layer with kernel size *K* = 16 and stride *S* = 8. The number of patches is:


$$\begin{array}{l} N=\left\lfloor \frac{T-K}{S}\right\rfloor +1 \end{array}$$ (3)

For *T* = 2500, this yields *N* = 311 patches. The embedding layer projects each patch into a *D*_token_ = 128 dimensional latent vector. The resulting token sequence is:


$$\begin{array}{l} \text{E}\in {\mathbb{R}}^{N\times D_{\text{token}}} \end{array}$$ (4)

Like ViT, the patch embeddings are linearly projected to the encoder's hidden dimension *D*_enc_ = 768 at the input of the encoder using a linear projection layer ${\text{W}}_{\text{proj}}\in {\mathbb{R}}^{768\times 128}$.

##### 3.2.1.2 Masking strategy

Masked Autoencoders (MAE) were adopted as the basis and 75% of all the EEG patches generated during the pre-training process were masked. The contiguous block masking and random patch masking was employed as a hybrid masking strategy to promote learning of local and long-range temporal dependencies. The binary mask vector is given by:


$$\begin{array}{l} \text{m}\in {\left\{0,1\right\}}^{N},\;\overset{N}{\underset{i=1}{\sum }}m_{i}=\left\lfloor 0.25N\right\rfloor \end{array}$$ (5)

where *m*_*i*_ = 1 indicates a visible (unmasked) patch. Specifically, 60% of masked patches were selected via contiguous block masking (randomly sized blocks of length 4–12 patches), and the remaining 40% were randomly sampled uniformly.

##### 3.2.1.3 Encoder-decoder architecture

The encoder consisted of a Vision Transformer (ViT)-style architecture with 12 transformer blocks, 12 attention heads, and an embedding dimension of *D*_enc_ = 768. Only visible EEG patches were processed by the encoder. Let **E**_vis_ be the subsequence of tokens with *m*_*i*_ = 1. The encoder maps them to latent representations:


$$\begin{array}{l} {\text{H}}_{\text{enc}}=\text{Encoder}\left({\text{E}}_{\text{vis}}\right),\;{\text{H}}_{\text{enc}}\in {\mathbb{R}}^{\left(1-0.75\right)N\times D_{\text{enc}}} \end{array}$$ (6)

A lightweight transformer decoder containing four layers and a hidden dimension of *D*_dec_ = 256 was employed to reconstruct the masked EEG patches. The decoder takes the encoded visible tokens concatenated with learned mask tokens for the masked positions:


$$\begin{array}{l} \hat{\text{X}}=\text{Decoder}\left(\left[{\text{H}}_{\text{enc}};{\text{M}}_{\text{learned}}\right]\right)\in {\mathbb{R}}^{N\times D_{\text{token}}} \end{array}$$ (7)

where **M**_learned_ are learnable mask tokens.

##### 3.2.1.4 Training objective

The overall self-supervised objective was defined as:


$$\begin{array}{l} {ℒ}_{\text{total}}={ℒ}_{\text{recon}}+\lambda {ℒ}_{\text{temp}} \end{array}$$ (8)

where ℒ_recon_ denotes the reconstruction loss computed only over masked patches using mean squared error (MSE):


$$\begin{array}{l} {ℒ}_{\text{recon}}=\frac{1}{\sum_{i=1}^{N}\left(1-m_{i}\right)}\underset{i:m_{i}=0}{\sum }\|{\hat{\text{e}}}_{i}-{\text{e}}_{i}{\|}_{2}^{2} \end{array}$$ (9)

Here, ${\text{e}}_{i}\in {\mathbb{R}}^{D_{\text{token}}}$ is the original embedded patch, and ${\hat{\text{e}}}_{i}$ is the reconstructed patch.

The temporal contrastive loss ℒ_temp_ drives the model to capture temporal relationships and sequence structure. In our framework, this is implemented as a temporal order prediction task where the model predicts whether neighboring patch pairs are in correct temporal order or swapped. The classifier predicts the probability *p*_*i,i*+1_, for each pair (*i, i* + 1), that the temporal order is correct:


$$\begin{array}{l} {ℒ}_{\text{temp}}=-\frac{1}{N_{\text{pairs}}}\underset{\left(i,i+1\right)}{\sum }\left[y_{i,i+1}\log p_{i,i+1}+\left(1-y_{i,i+1}\right)\log\left(1-p_{i,i+1}\right)\right] \end{array}$$ (10)

The values of *y*_*i,i*+1_ are 1 if the order is correct and 0 otherwise. This forces the model to learn about the temporal ordering of EEG signals, which is essential to capture the temporal nature of the neural activity. The loss can be decomposed into reconstruction loss (ℒ_recon_) and temporal contrastive loss (ℒ_temp_), which is given with a weight of 0.1.

#### 3.2.2 Pretraining configuration

The hyperparameters listed in Table 2 were used to pretrain the SSL framework on the SRM dataset (OpenNeuro ds003775). The optimization of the model was done by using the AdamW optimizer and cosine learning rate scheduling. About 48 h of pretraining was done on an NVIDIA A100 GPU.

**Table 2:** Self-supervised pretraining configuration.

Parameter	Value
Optimizer	AdamW
Learning rate	1 × 10^−4^
Batch size	32
Epochs	200
Weight decay	0.05
Dropout	0.1
Gradient clipping	1.0
Warm-up steps	1,000

### 3.3 Proposed spatio-temporal transformer architecture

#### 3.3.1 STT-EEG overview

**Figure 3:**
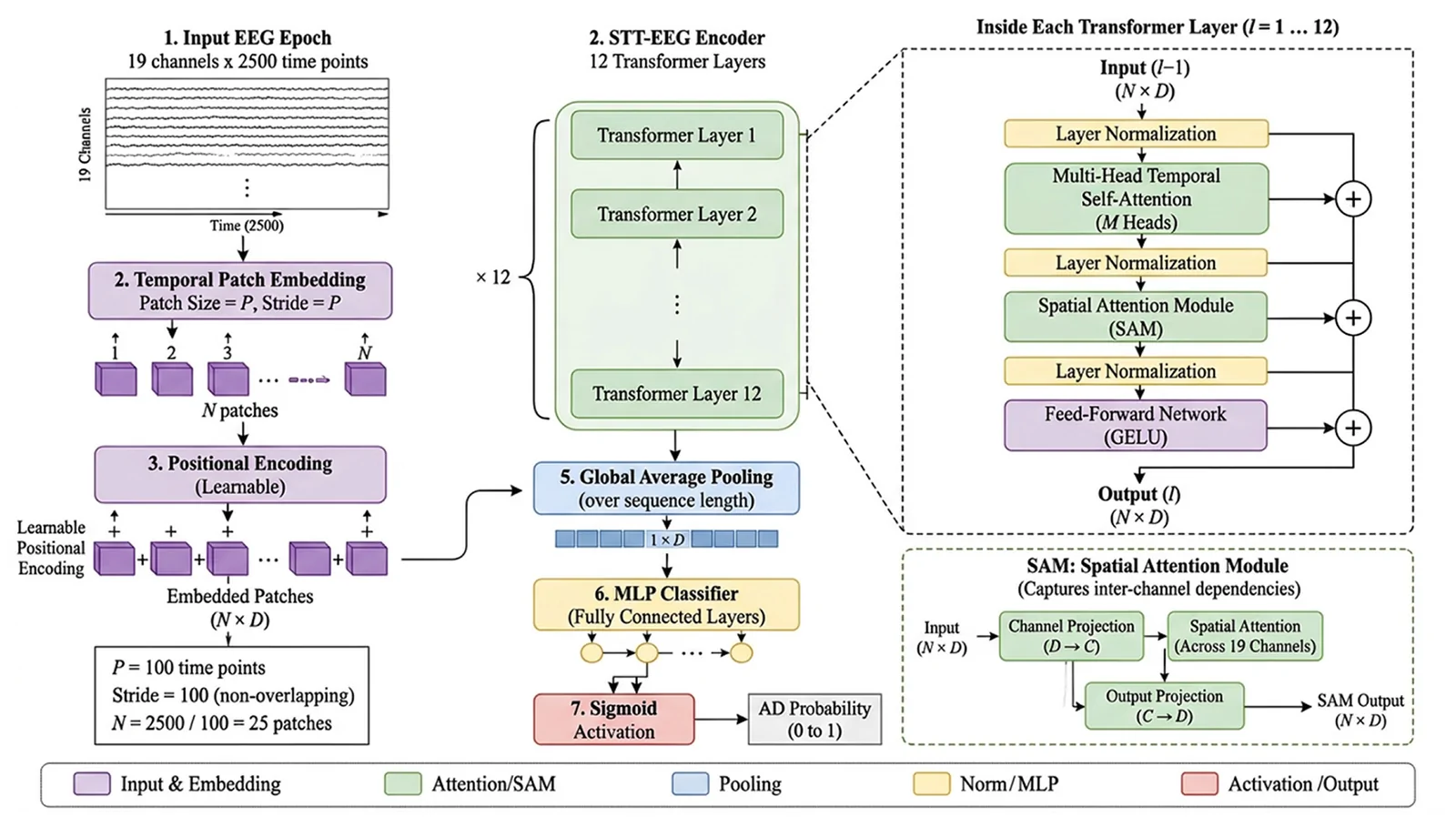
Architecture of the proposed spatio-temporal transformer for EEG (STT-EEG). The input EEG epoch **X** ∈ ℝ^19×2500^ is projected into a latent representation with spatial embedding and positional encoding. The Transformer encoder integrates temporal self-attention, spatial attention, and feed-forward networks with residual connections and layer normalization. A global average pooling layer generates the feature representation **h** ∈ ℝ^768^, which is classified by a two-layer MLP with sigmoid activation for Alzheimer's disease probability prediction.

This research presents Spatio-Temporal Transformer for EEG (STT-EEG) architecture that was developed for modeling both temporal dynamics and spatial interactions among the EEG channels. The overall framework starts by dividing the continuous EEG signal into non-overlapping temporal patches into a sequence of latent representations using a temporal patch embedding layer. These patch embeddings are then combined with learnable positional encodings to maintain the temporal hierarchy of the EEG signal. The backbone of the architecture consists of a stack of transformer layers that include two complementary attention mechanisms: a temporal self-attention module with multiple heads that models long-range temporal dependencies, and a spatial attention module that explicitly models cross-channel interactions to learn functional connectivity patterns that are relevant to Alzheimer's disease. A feed-forward network is used to refine the features pointwise after every attention block and layer normalization and residual connections are used to ensure training stability and gradient flow. The last one is a classification head which combines all the learned spatio-temporal representations by global average pooling, and then passes them through a two-layer multilayer perceptron with sigmoid activation to get the AD probability score. The unified design allows the STT-EEG to capitalize on both the temporal structure of resting state dynamics and spatial topology of electrode configuration on the scalp. The complete architecture of the proposed Spatio-Temporal Transformer for EEG (STT-EEG) is illustrated in Figure 3.

#### 3.3.2 Input embedding

Given an EEG epoch **X** ∈ ℝ^*C*×*T*^, we first apply a learnable linear projection to each time point to obtain a spatial embedding:


$$\begin{array}{l} \text{S}\left(t\right)={\text{W}}_{s}\text{x}\left(t\right)+{\text{b}}_{s},\;t=1,\ldots ,T \end{array}$$ (11)

where **x**(*t*) ∈ ℝ^*C*^ denotes the vector of all channels at time *t*, ${\text{W}}_{s}\in {\mathbb{R}}^{D_{\text{model}}\times C}$ is a learnable weight matrix, ${\text{b}}_{s}\in {\mathbb{R}}^{D_{\text{model}}}$ is a bias term, and *D*_model_ = 768 is the model's hidden dimension. Thus, $\text{S}\left(t\right)\in {\mathbb{R}}^{D_{\text{model}}}$ represents the spatially mixed signal at time *t*. The sequence $\text{S}\in {\mathbb{R}}^{T\times D_{\text{model}}}$ is then augmented with learned positional embeddings to preserve temporal order:


$$\begin{array}{l} {\text{Z}}^{\left(0\right)}=\text{S}+\text{P},\;\text{P}\in {\mathbb{R}}^{T\times D_{\text{model}}} \end{array}$$ (12)

where **P** is a learnable positional encoding matrix.

#### 3.3.3 Spatio-temporal attention encoder

The transformer encoder consisted of *L* = 12 transformer layers. For each layer *l* there was multi-head temporal self-attention (MSA), a spatial attention module (SAM), and a feed-forward network (FFN), followed by layer normalization (LN) and residual connections.

##### 3.3.3.1 Temporal multi-head self-attention

The temporal self-attention module is responsible for modeling long-range temporal dependencies within the EEG sequence. Unlike recurrent architectures that process signals sequentially, the transformer-based temporal attention mechanism enables the network to directly capture relationships between distant time steps in parallel. This is particularly important for EEG-based Alzheimer's disease (AD) analysis, where pathological neural patterns may appear intermittently across different temporal segments of the recording. Assume the input to the *l*-th transformer block is represented as:


$$\begin{array}{l} {\text{Z}}^{\left(l-1\right)}\in {\mathbb{R}}^{T\times D_{\text{model}}} \end{array}$$ (13)

where *T* denotes the temporal length and *D*_model_ represents the embedding dimension. First, layer normalization is applied:


$$\begin{array}{l} {\text{A}}^{\left(l\right)}=\text{LN}\left({\text{Z}}^{\left(l-1\right)}\right) \end{array}$$ (14)

The normalized embeddings are then linearly projected into query, key, and value spaces:


$$\begin{array}{l} \text{Q}={\text{A}}^{\left(l\right)}{\text{W}}_{Q},\;\text{K}={\text{A}}^{\left(l\right)}{\text{W}}_{K},\;\text{V}={\text{A}}^{\left(l\right)}{\text{W}}_{V} \end{array}$$ (15)

where **W**_*Q*_, **W**_*K*_, and **W**_*V*_ are learnable projection matrices.

The temporal MSA is computed as:


$$\begin{array}{l} {\text{T}}^{\left(l\right)}=\text{MSA}\left(\text{Q},\text{K},\text{V}\right)+{\text{Z}}^{\left(l-1\right)} \end{array}$$ (16)

The attention mechanism computes pairwise temporal correlations using scaled dot-product attention:


$$\begin{array}{l} \text{Attention}\left(\text{Q},\text{K},\text{V}\right)=\text{Softmax}\left(\frac{\text{Q}{\text{K}}^{⊤}}{\sqrt{d_{k}}}\right)\text{V} \end{array}$$ (17)

where *d*_*k*_ denotes the dimensionality of the key vectors. Multi-head attention enables the model to jointly attend to multiple temporal subspaces and capture diverse neural dynamics simultaneously.

This operation captures temporal dependencies across all time steps while preserving contextual information through residual connections.

##### 3.3.3.2 Spatial attention module (SAM)

**Figure 4:**
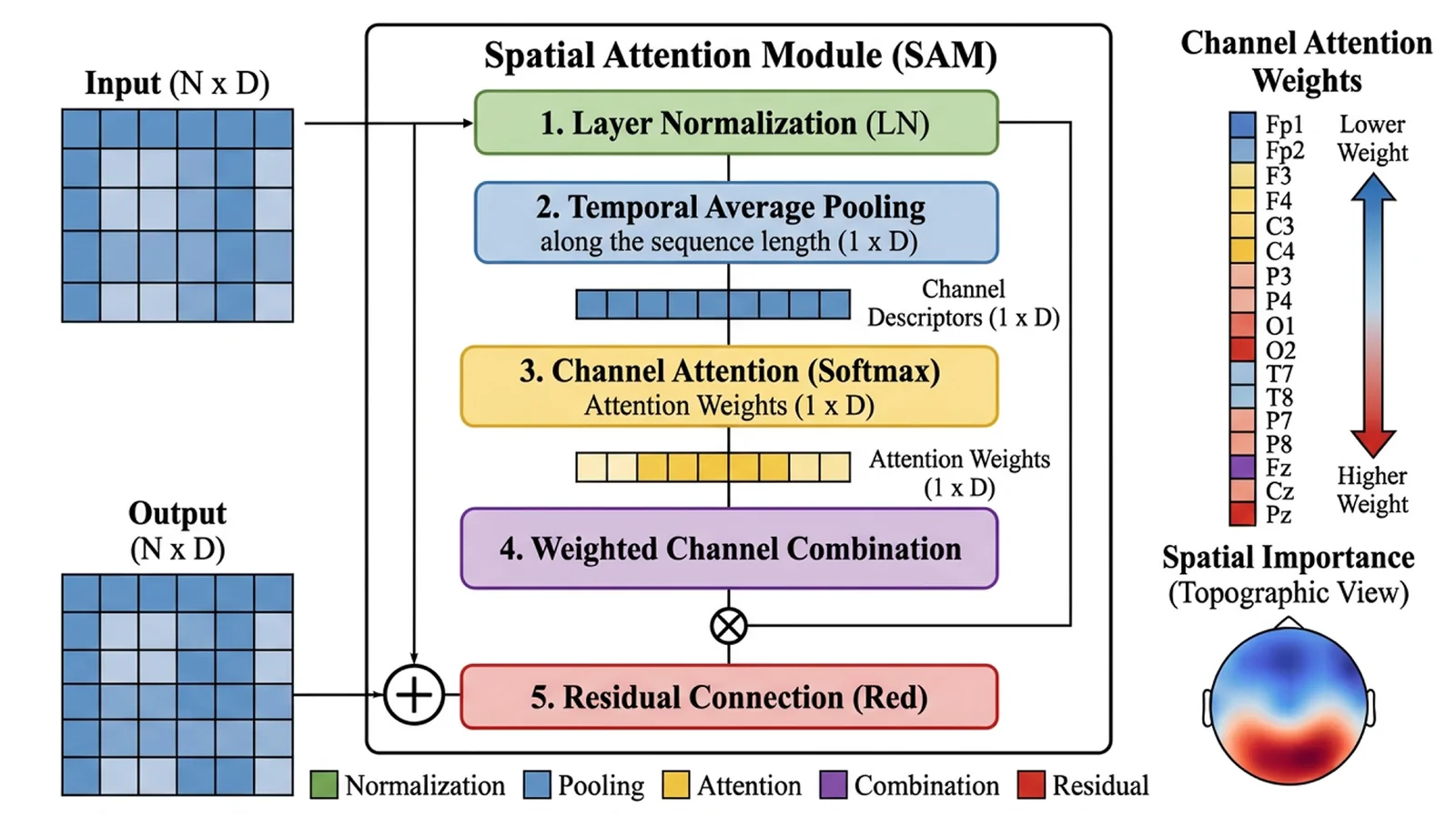
Detailed illustration of the proposed Spatial Attention Module (SAM). The temporal self-attention output is normalized and aggregated through temporal pooling to generate channel-wise attention weights via a learnable projection and softmax operation. The weighted channel representation highlights discriminative EEG regions while suppressing irrelevant information through a residual connection.

In addition to temporal attention, the EEG signals have strong inter-channel correlations as well, which are the result of functional connectivity among different brain regions. To explicitly model the interactions between channels and improve the discriminative spatial representations, a dedicated module called the Spatial Attention Module (SAM) was added. The detailed architecture of the proposed Spatial Attention Module (SAM) for identifying discriminative brain regions is illustrated in Figure 4. Then another layer normalization operation is carried out:


$$\begin{array}{l} {\text{B}}^{\left(l\right)}=\text{LN}\left({\text{T}}^{\left(l\right)}\right) \end{array}$$ (18)

To aggregate global temporal information for each channel, average pooling is applied across the temporal dimension:


$$\begin{array}{l} {\bar{\text{B}}}^{\left(l\right)}=\frac{1}{T}\overset{T}{\underset{t=1}{\sum }}{\text{B}}_{t,:}^{\left(l\right)}\in {\mathbb{R}}^{D_{\text{model}}} \end{array}$$ (19)

This operation summarizes the overall contribution of each feature dimension over the complete EEG sequence. Spatial attention weights are then computed using a learnable projection matrix:


$$\begin{array}{l} \alpha^{\left(l\right)}=\text{Softmax}\left({\bar{\text{B}}}^{\left(l\right)}{\text{W}}_{\text{spatial}}\right) \end{array}$$ (20)

where ${\text{W}}_{\text{spatial}}\in {\mathbb{R}}^{D_{\text{model}}\times C}$ projects the latent representation into the channel-attention space, and *C* denotes the number of EEG channels.

The resulting attention coefficients $\alpha_{c}^{\left(l\right)}$ quantify the relative importance of each EEG channel for downstream AD classification. The attended representation is formulated as:


$$\begin{array}{l} {\text{C}}^{\left(l\right)}=\overset{C}{\underset{c=1}{\sum }}\alpha_{c}^{\left(l\right)}{\text{B}}_{:,c}^{\left(l\right)}+{\text{T}}^{\left(l\right)} \end{array}$$ (21)

Through this mechanism, the network adaptively emphasizes informative brain regions while suppressing redundant or noisy channels, thereby improving spatial feature discrimination.

In the SAM, the temporal average pooling is performed before calculating spatial attention weights. Temporal average pooling is performed prior to computing spatial attention weights in the SAM. This is an intentional design choice with three key justifications: (1) SAM aims to detect the spatially most important channels not for the entire 10-s epoch but rather for the entire EEG recording. The goal of SAM is to identify which EEG channels are globally important for classification of ADs, rather than to capture moment-by-moment spatial dynamics. The pathological changes in AD can be detected as chronic alterations in oscillatory dynamics (slowing, reduced complexity, disrupted connectivity) throughout the entire resting-state recording. (2) The Temporal multi-head self-attention (TMHSA) module explicitly considers time-varying patterns across the sequence. SAM fills this in by incorporating a global spatial weighting emphasizing channels of overall relevance. Temporal attention identifies the temporal patterns, and SAM identifies the spatial patterns. (3) By computing temporal averages over the whole 10-second window, instead of possibly noisy time points, the resulting spatial attention estimates will be more stable and efficient.

##### 3.3.3.3 Feed-forward network

Temporal and spatial attention refinement is followed by using a position-wise feed-forward network (FFN) to further strengthen the capability of non-linear feature transformation. Each temporal token is processed independently by the FFN with shared fully connected layers. Then, layer normalization is used:


$$\begin{array}{l} {\text{Z}}^{\left(l\right)}=\text{FFN}\left(\text{LN}\left({\text{C}}^{\left(l\right)}\right)\right)+{\text{C}}^{\left(l\right)} \end{array}$$ (22)

The FFN consists of two linear transformations separated by a Gaussian Error Linear Unit (GELU) activation function:


$$\begin{array}{l} \text{FFN}\left(\text{u}\right)={\text{W}}_{2}\cdot \text{GELU}\left({\text{W}}_{1}\text{u}+{\text{b}}_{1}\right)+{\text{b}}_{2} \end{array}$$ (23)

where **W**_1_ and **W**_2_ are learnable weight matrices, and **b**_1_ and **b**_2_ denote bias terms.

Compared with conventional ReLU activations, GELU provides smoother nonlinear transitions and has demonstrated superior optimization stability in transformer architectures. The complete transformer operations are summarized as follows:


$$\begin{array}{l} {{\text{Z}}^{\prime }}^{\left(l\right)}=\text{MSA}(\text{LN}({\text{Z}}^{\left(l-1\right)}))+{\text{Z}}^{\left(l-1\right)} \end{array}$$ (24)

$$\begin{array}{l} {{\text{Z}}^{\prime \prime }}^{\left(l\right)}=\text{SAM}(\text{LN}({{\text{Z}}^{\prime }}^{\left(l\right)}))+{{\text{Z}}^{\prime }}^{\left(l\right)} \end{array}$$ (25)

$$\begin{array}{l} {\text{Z}}^{\left(l\right)}=\text{FFN}(\text{LN}({{\text{Z}}^{\prime \prime }}^{\left(l\right)}))+{{\text{Z}}^{\prime \prime }}^{\left(l\right)} \end{array}$$ (26)

The combination of temporal attention, spatial attention, and nonlinear feed-forward transformation enables the proposed STT-EEG architecture to jointly learn discriminative spatio-temporal EEG representations for robust AD prediction.

#### 3.3.4 Classification head

After passing through *L* transformer layers, the final latent representation **Z**^(*L*)^ contains enriched spatio-temporal information derived from both temporal dependencies and inter-channel interactions. To obtain a compact global representation, temporal global average pooling is applied:


$$\begin{array}{l} \text{h}=\frac{1}{T}\overset{T}{\underset{t=1}{\sum }}{\text{Z}}_{t,:}^{\left(L\right)}\in {\mathbb{R}}^{D_{\text{model}}} \end{array}$$ (27)

This pooling strategy reduces the temporal dimension while preserving the overall contextual information across the EEG sequence. The aggregated feature vector is subsequently passed through a two-layer multilayer perceptron (MLP) classifier:


$$\begin{array}{l} ŷ=\sigma \left({\text{W}}_{\text{out}}^{\left(2\right)}\cdot \text{ReLU}\left({\text{W}}_{\text{out}}^{\left(1\right)}\text{h}+{\text{b}}_{\text{out}}^{\left(1\right)}\right)+{\text{b}}_{\text{out}}^{\left(2\right)}\right) \end{array}$$ (28)

where ${\text{W}}_{\text{out}}^{\left(1\right)}$ and ${\text{W}}_{\text{out}}^{\left(2\right)}$ denote learnable weight matrices, while ${\text{b}}_{\text{out}}^{\left(1\right)}$ and ${\text{b}}_{\text{out}}^{\left(2\right)}$ are bias vectors.

The sigmoid activation function σ(·) converts the network output into a probability score for binary AD classification.

### 3.4 Fine-tuning strategy

The pretrained STT-EEG framework was fine-tuned with the transfer learning approach using 5-fold cross-validation with stratification by subject to adapt it for Alzheimer's disease prediction. A subject independent partitioning strategy was strictly followed so as to avoid leaking information between training and testing sets. In particular, the eeg epochs were only assigned to a single partition, if they came from the same subject. The spatio-temporal representations learned by the pretrained transformer backbone were maintained by using self-supervised weights and optimizing with a smaller learning rate during training.


$$\begin{array}{l} \eta_{\text{backbone}}=1\times 10^{-5} \end{array}$$ (29)

In contrast, the newly initialized classification head was trained using a larger learning rate:


$$\begin{array}{l} \eta_{\text{classifier}}=1\times 10^{-3} \end{array}$$ (30)

This differential learning strategy stabilizes optimization while enabling rapid adaptation of the classification layers. To mitigate class imbalance between AD and cognitively normal (CN) subjects, weighted binary cross-entropy loss was employed:


$$\begin{array}{l} {ℒ}_{\text{BCE}}=-\frac{1}{N_{\text{batch}}}\overset{N_{\text{batch}}}{\underset{i=1}{\sum }}\left[w\cdot y_{i}\log{ŷ}_{i}+\left(1-y_{i}\right)\log\left(1-{ŷ}_{i}\right)\right] \end{array}$$ (31)

where:


$$\begin{array}{l} w=\frac{N_{\text{CN}}}{N_{\text{AD}}} \end{array}$$ (32)

denotes the inverse class-frequency weighting factor.

The AdamW optimizer was utilized for parameter optimization due to its improved regularization capability and stable convergence behavior in transformer-based networks. Furthermore, early stopping with a patience of 20 epochs was adopted to reduce overfitting and improve model generalization performance.

### 3.5 Interpretability analysis

To enhance clinical interpretability, we incorporated attention-based explainability mechanisms.

#### 3.5.1 Attention rollout

Attention rollout was employed to visualize the contribution of EEG channels and temporal regions to the final model prediction. Let ${\text{A}}_{h}^{\left(l\right)}$ be the attention matrix for head *h* in layer *l*. The aggregated attention from input to output is:


$$\begin{array}{l} \tilde{\text{A}}=\overset{L}{\underset{l=1}{\prod }}\left(\frac{1}{H}\overset{H}{\underset{h=1}{\sum }}{\text{A}}_{h}^{\left(l\right)}+\text{I}\right) \end{array}$$ (33)

where **I** is the identity matrix to account for residual connections. The resulting matrix provides spatio-temporal importance maps.

#### 3.5.2 Channel perturbation analysis

A leave-one-channel-out perturbation strategy was further used to assess channel importance. Individual EEG channels were systematically suppressed, and the resulting decrease in classification performance was measured:


$$\begin{array}{l} \text{Importance}\left(c\right)={\text{AUC}}_{\text{full}}-{\text{AUC}}_{\text{without }c} \end{array}$$ (34)

## 4 Experimental configuration

### 4.1 Experimental setup

The experiments were all carried out in Python 3.10, with the deep learning framework PyTorch 2.0, EEG data processing with MNE-Python, and artifact correction and preprocessing with EEGLAB. All proposed baseline models and the Spatio-Temporal Transformer for EEG (STT-EEG) were trained and tested on an NVIDIA A100 GPU with 40 GB of memory, allowing for efficient computation of the transformer's multi-head self-attention mechanisms and large-scale matrix operations required in the processing of EEG epochs. The STT-EEG encoder was set up with *L* = 12 transformer layers, *H* = 12 attention heads, and a hidden dimension of *D*_model_ = 768 as detailed in Table 2. To achieve a lightweight design in the decoder side, the number of transformer layers was limited to four in this work and the hidden dimension was decreased to 256 to lower computational costs during reconstruction. When fine-tuning for Alzheimer's disease classification, same architecture for the encoder and randomly initialized classification head. In all experiments the AdamW optimizer was employed with a weight decay of 0.05 and gradient clipping of 1.0 to avoid gradient explosion. Both pretraining and fine-tuning were carried out with a constant batch size of 32.

The 65 subjects (36 AD, 29 CN) were randomly split into 5 folds, with stratification by class (AD/CN) to ensure the same number of subjects in each fold. In particular, the respective scikit-learn StratifiedKFold was used with shuffle=True and random_state=42. There were around 13 subjects (7–8 AD, 5–6 CN) in each fold. Each EEG epoch within a subject was only applied to his or her specific fold. There was no subject that was used in more than one fold of the 5-fold CV. We used a strict subject-wise stratified 5-fold cross validation approach to avoid data leakage. The data were augmented on-the-fly during training, but not stored or shared among folds. The validation and test sets were preserved to make sure that the performance is unbiased. The very strict artifact rejection pipeline (ASR, ICA with ICLabel) ensures that there is a low risk of biases at the session or dataset level. The learning rate of the pretrained transformer backbone was set to be smaller, 1 × 10^−5^, and the learning rate of the classification head was set to be larger, 1 × 10^−3^. The experiments were performed five times with different random seeds to obtain statistical confidence; the mean ± SD is reported.

### 4.2 Data augmentation

Deep learning models, particularly transformer-based models, are very parameterized and prone to over-fitting on small datasets. To reduce the risk and enhance robustness, generalization, and invariance to physiologically irrelevant variations, we only used a set of EEG-specific data augmentation techniques on-the-fly during the fine-tuning phase on the OpenNeuro ds004504 dataset (65 subjects). Each of these augmentations were carefully chosen to retain the pathological properties of the Alzheimer's disease while simultaneously adding realistic variation to represent real-world recording scenarios and biological variation. For all augmentations probabilities of 0.5 were applied only during the training phase, while the validation and test sets remained unchanged to avoid any bias in the evaluation of the performance.

**Figure 5:**
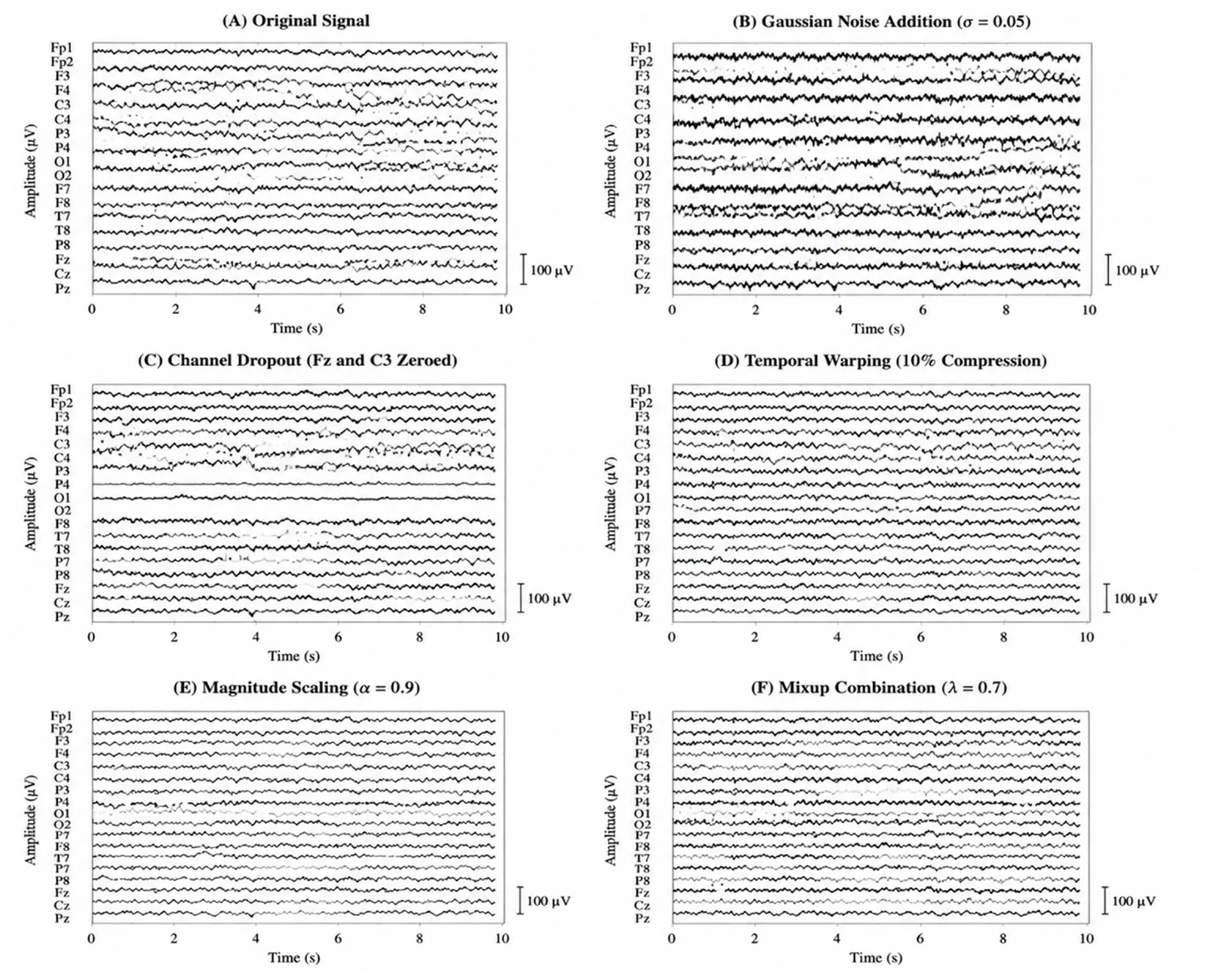
Data augmentation strategies for EEG data applied on a representative 10 s AD EEG segment. The augmentations are as follows: Gaussian noise addition, channel dropout, temporal warping, magnitude scaling and Mixup. These transformations allow for inclusion of realistic variations in the signal while maintaining important oscillatory and pathological features. Only augmentations were used during training.

Self-supervised pretraining was done without data augmentation, to ensure that the model sees the natural distribution of EEG signals from healthy controls. The problem is that if the signal statistics are changed during the pretraining, the model may learn the wrong signal statistics as its “base” distribution. In fine tuning, however, the purpose of data augmentation is different: it introduces realistic recording variability for fine tuning the model, whilst maintaining pathological features without compromising the model's ability to learn from the small number of labeled AD cases (n=65 subjects). This strategic design follows the idea that the pretraining should learn the distribution of the data generated, while the fine-tuning should learn the task-discriminating features robustly. The independent validation set (OpenNeuro ds004148) was also evaluated without augmentation to preserve unbiased performance assessment. Examples of EEG-specific data augmentations applied to a representative 10-s AD patient segment are illustrated in Figure 5.

#### 4.2.1 Gaussian noise addition

Electroencephalographic recordings are inherently susceptible to various noise sources, including thermal noise from amplifiers, electromagnetic interference, and physiological artifacts not fully removed during preprocessing. To simulate such real-world conditions and improve model robustness to signal corruption, we added Gaussian noise to the raw EEG signals.

For each epoch **X** ∈ ℝ^*C*×*T*^, where *C* = 19 channels and *T* = 2, 500 time points, the augmented signal $\tilde{\text{X}}$ was computed as:


$$\begin{array}{l} \tilde{\text{X}}=\text{X}+\epsilon ,\;\epsilon \sim 𝒩\left(0,\sigma^{2}\cdot \text{Var}\left(\text{X}\right)\right) \end{array}$$ (35)

The noise level relative to signal variance is controlled by σ = 0.05, yielding a signal-to-noise ratio (SNR) of approximately 20 dB. This causes detectable but not overwhelming perturbations, helping the model generalize to recordings with different noise properties.

#### 4.2.2 Channel dropout

Poor electrode contact can occur intermittently during EEG recording, causing brief signal loss or distortion in one or several channels. Simulating this phenomenon forces the model to learn redundant and spatially distributed representations. For each channel, the dropout probability was set to *p*_drop_ = 0.10 (approximately 2 out of 19 channels). For each selected channel *c*, the entire time series was set to zero:


$$\begin{array}{l} {\tilde{\text{X}}}_{c,:}=0^{⊤},\;c\sim \text{Bernoulli}\left(p_{\text{drop}}\right) \end{array}$$ (36)

Channel dropout encourages the model to infer missing information from remaining channels, which is particularly relevant for AD where pathological changes are distributed across the network rather than isolated to specific areas.

#### 4.2.3 Temporal warping

Natural fluctuations in oscillatory rhythms due to changes in arousal, attention, and vigilance introduce physiological variability. Temporal warping simulates this variability through smooth, non-uniform time dilations and compressions. We implemented a modified version using random sub-segment stretching/compression by 10% on each side of the epoch, with linear interpolation to preserve the sampling rate:


$$\begin{array}{l} {\tilde{\text{X}}}_{c,t}={\text{X}}_{c,\psi \left(t\right)},\;\psi \left(t\right)=t+\eta \cdot \sin\left(\frac{2\pi t}{T}\cdot \kappa \right) \end{array}$$ (37)

where η ~ 𝒰(−10, 10) controls warp magnitude (in samples), and κ ~ 𝒰(1, 5) controls warp frequency. Temporal warping enhances model invariance to rate changes in neural oscillations that are clinically irrelevant for AD diagnosis.

#### 4.2.4 Magnitude scaling

EEG signal amplitudes vary across individuals due to anatomical differences (skull thickness, cortical folding), electrode impedance variations, and instrument calibration differences. To simulate this variability, we applied random magnitude scaling to each epoch:


$$\begin{array}{l} \tilde{\text{X}}=\alpha \cdot \text{X},\;\alpha \sim 𝒰\left(0.8,1.2\right) \end{array}$$ (38)

where α is drawn independently for each epoch. This prevents the model from relying on absolute amplitude cues, which are highly variable across recordings, and instead focuses on relative amplitude patterns and oscillatory dynamics informative of AD pathology.

#### 4.2.5 Mixup augmentation

To address limited labeled data and class imbalance in our dataset (36 AD vs. 29 CN), we applied Mixup (Zhang et al., 2017), a data-dependent augmentation technique that generates convex combinations of pairs of training examples and their corresponding labels. Given two randomly sampled epochs (**X**_*i*_, *y*_*i*_) and (**X**_*j*_, *y*_*j*_), where *y* ∈ {0, 1} indicates AD (1) or CN (0), the mixed epoch $\tilde{\text{X}}$ and mixed label ỹ were computed as:


$$\begin{array}{l} \tilde{\text{X}}=\lambda {\text{X}}_{i}+\left(1-\lambda \right){\text{X}}_{j} \end{array}$$ (39)

$$\begin{array}{l} ỹ=\lambda y_{i}+\left(1-\lambda \right)y_{j} \end{array}$$ (40)

where λ ~ Beta(α_mix_, α_mix_) with α_mix_ = 0.2. The Beta distribution concentrates probability mass near 0 and 1, producing mixtures that remain close to original examples while interpolating between class boundaries.

To ensure pathological coherence and ensure biologically implausible mixtures between AD and CN signals were not generated, mixup was only applied to pairs within the same class. While in image classification the mixing of the classes could be regarded as semantically meaningful blends, in the case of pathological and normal EEG signals, the mixing could lead to a fundamental alteration of the underlying neural dynamics which could not be considered to be physiologically meaningful. The oscillatory signatures of AD (decreased alpha power, increased delta/theta activity, reduced complexity) are true markers of pathological changes, and do not simply slide between normal signatures along a linear continuum. This design decision was validated empirically, as same-class Mixup was more successful in our pilot experiments, maintaining the pathological coherence of AD signals while also affording regularization benefits. The Mixup method increases the size of the training set by a factor of two and more, which helps to reduce overfitting, and introduces linear relationships between training samples, resulting in better model calibration and generalization (Thulasidasan et al., 2019).

#### 4.2.6 Augmentation pipeline and hyperparameters

All augmentations were applied sequentially during training, each independently activated with probability 0.5. The fixed order was: Gaussian noise → channel dropout → temporal warping → magnitude scaling → Mixup.

#### 4.2.7 Rationale for augmentation selection

We selected these augmentation techniques based on three criteria: (1) physiological plausibility: all augmentations mimic real-world variability observed in clinical EEG recordings; (2) label preservation: augmentations do not alter the pathological status of the signal; and (3) empirical effectiveness: each technique has proven useful in previous EEG deep learning studies (Lashgari et al., 2020; Mohsenvand et al., 2019). We deliberately avoided augmentations that would interfere with AD-relevant features. Frequency filtering was not used because decreased alpha power and increased delta/theta power are key pathological signatures of AD. Channel swapping and EEG source mixing were omitted to preserve spatial connectivity patterns crucial for AD detection.

### 4.3 Evaluation metrics

The performance of the proposed self-supervised spatio-temporal transformer (STT-EEG) was evaluated using a comprehensive set of classification and regression metrics. These metrics were selected to assess both the primary task of Alzheimer's disease (AD) classification and the secondary task of Mini-Mental State Examination (MMSE) score prediction. For the binary classification task (AD vs. cognitively normal (CN)), the evaluation was based on the confusion matrix components, including true positives (TP), true negatives (TN), false positives (FP), and false negatives (FN). To measure the overall classification performance, accuracy was computed as the ratio of correctly predicted samples to the total number of samples:


$$\begin{array}{l} \text{Accuracy}=\frac{TP+TN}{TP+TN+FP+FN} \end{array}$$ (41)

Since accuracy alone may provide misleading results in imbalanced datasets, additional metrics were considered. Sensitivity (Recall) was used to evaluate the ability of the model to correctly identify AD patients, which is particularly important in clinical diagnosis where missed detections can delay treatment:


$$\begin{array}{l} \text{Sensitivity}=\frac{TP}{TP+FN} \end{array}$$ (42)

Specificity was employed to assess the model's capability to correctly recognize cognitively normal subjects and reduce false alarms:


$$\begin{array}{l} \text{Specificity}=\frac{TN}{TN+FP} \end{array}$$ (43)

Precision measures the proportion of correctly predicted AD cases among all samples classified as AD:


$$\begin{array}{l} \text{Precision}=\frac{TP}{TP+FP} \end{array}$$ (44)

To balance both precision and recall, the F1-score was also calculated as their harmonic mean:


$$\begin{array}{l} \text{F1-Score}=2\times \frac{\text{Precision}\times \text{Sensitivity}}{\text{Precision}+\text{Sensitivity}} \end{array}$$ (45)

Balanced Accuracy was further included to provide a fair assessment under class imbalance conditions by averaging sensitivity and specificity:


$$\begin{array}{l} \text{Balanced Accuracy}=\frac{\text{Sensitivity}+\text{Specificity}}{2} \end{array}$$ (46)

To evaluate the discriminative capability of the proposed framework across different classification thresholds, the Area Under the Receiver Operating Characteristic Curve (AUC-ROC) was computed. AUC values closer to 1 indicate stronger discriminative performance:


$$\begin{array}{l} \text{AUC}=\int_{0}^{1}\text{TPR}\left(t\right)d\left(\text{FPR}\left(t\right)\right) \end{array}$$ (47)

where TPR(*t*) and FPR(*t*) denote the true positive rate and false positive rate at threshold *t*, respectively. In addition, the Matthews Correlation Coefficient (MCC) was used as a robust metric for imbalanced binary classification tasks because it incorporates all confusion matrix categories:


$$\begin{array}{l} \text{MCC}=\frac{TP\times TN-FP\times FN}{\sqrt{\left(TP+FP\right)\left(TP+FN\right)\left(TN+FP\right)\left(TN+FN\right)}} \end{array}$$ (48)

For the MMSE regression task, several standard regression metrics were employed to evaluate prediction accuracy and correlation. The Mean Absolute Error (MAE) was used to measure the average absolute difference between predicted and actual MMSE scores:


$$\begin{array}{l} \text{MAE}=\frac{1}{n}\overset{n}{\underset{i=1}{\sum }}|y_{i}-{ŷ}_{i}| \end{array}$$ (49)

where *y*_*i*_ and ŷ_*i*_ represent the actual and predicted MMSE scores, respectively. The Root Mean Squared Error (RMSE) was also computed to penalize larger prediction errors more strongly:


$$\begin{array}{l} \text{RMSE}=\sqrt{\frac{1}{n}\overset{n}{\underset{i=1}{\sum }}{\left(y_{i}-{ŷ}_{i}\right)}^{2}} \end{array}$$ (50)

To assess the linear relationship between predicted and actual MMSE scores, the Pearson correlation coefficient (*r*) was calculated:


$$\begin{array}{l} r=\frac{\sum_{i=1}^{n}\left(y_{i}-ȳ\right)\left({ŷ}_{i}-\bar{ŷ}\right)}{\sqrt{\sum_{i=1}^{n}{\left(y_{i}-ȳ\right)}^{2}}\sqrt{\sum_{i=1}^{n}{\left({ŷ}_{i}-\bar{ŷ}\right)}^{2}}} \end{array}$$ (51)

Finally, the coefficient of determination (*R*^2^) was employed to quantify the proportion of variance in MMSE scores explained by the model:


$$\begin{array}{l} R^{2}=1-\frac{\sum_{i=1}^{n}{\left(y_{i}-{ŷ}_{i}\right)}^{2}}{\sum_{i=1}^{n}{\left(y_{i}-ȳ\right)}^{2}} \end{array}$$ (52)

All evaluation metrics were reported as mean ± standard deviation across five-fold subject-wise cross-validation.

### 4.4 Baseline methods

To comprehensively evaluate the effectiveness of the proposed STT-EEG framework, comparisons were conducted against a diverse set of baseline approaches, including traditional handcrafted feature-based methods, supervised deep learning architectures, and recently developed EEG foundation models.

Among conventional machine learning approaches, Chedid et al. (2022) proposed an automated machine learning framework that integrates statistical, spectral, and functional connectivity features with optimized ensemble classifiers for EEG-based neurological analysis. Similarly, Miltiadous et al. (2023b) utilized the OpenNeuro ds004504 dataset and combined spectral power, complexity, and synchronization features with random forest classifiers, achieving 95.86% accuracy for predicting cognitive decline in amnestic mild cognitive impairment (aMCI) patients after one year. In another study, Babiloni et al. (2026) employed eLORETA-based source localization and multiple machine learning classifiers for cognitive decline prediction in aMCI subjects, reporting an accuracy of 76.60%. Furthermore, Deng et al. (2024) introduced a multiscale functional connectivity framework using wavelet decomposition and ensemble AdaBoost classifiers for mild cognitive impairment (MCI) detection on a cohort of 137 elderly participants. To provide broader neuroimaging comparisons, two additional state-of-the-art methods were considered. Li et al. (2026) achieved an AUC of 94.00% for Alzheimer's disease (AD) versus cognitively normal (CN) classification using structural MRI-based brain atrophy scoring, while Ahamed et al. (2025) reported 99.45% accuracy for four-class AD classification using a modified EfficientNetV2B3 architecture trained on hybrid-filtered MRI images.

In addition to conventional approaches, several advanced deep learning models for EEG and time-series analysis were included as baselines. Zhang et al. (2021) introduced EEGInception, a multi-scale convolutional neural network (CNN) architecture with parallel convolutional kernels of varying lengths to capture diverse temporal patterns in EEG signals. Song et al. (2022) proposed EEGConformer, which combines CNN-based feature extraction with Transformer-based temporal-spatial self-attention mechanisms to improve long-range dependency modeling. Bai et al. (2018) developed Temporal Convolutional Networks (TCN) using dilated causal convolutions for efficient long-sequence modeling, whereas Nie et al. (2023) introduced PatchTST, a patch-based Transformer framework that segments time series into patches and processes channels independently for scalable sequence learning. Wang et al. (2025) proposed Medformer, a multi-granularity patching Transformer designed to capture intra-period and inter-period variations in medical time-series data. Finally, Wang et al. (2024) proposed CBraMod (Cross-brain Foundation Model), a large-scale EEG foundation model based on a criss-cross attention architecture. CBraMod was pre-trained on approximately 2,500 hours of heterogeneous brain signals collected from multiple EEG acquisition settings and demonstrated state-of-the-art transfer learning performance across diverse brain signal processing tasks.

Furthermore, Jiang et al. (2024) designed LaBRaM (Large Brain Model), a foundation model for EEG, which is pretrained using masked autoencoding on massive EEG data such as TUH EEG, Sleep EDF, and motor imagery data. The transformer-based architecture pretrained with masked token prediction provides generic representations learned from tremendous EEG data, and transfer learning with LaBraM achieves state-of-the-art results across different EEG classification benchmarks.

#### 4.4.1 Implementation details

In order to obtain a fair and reproducible comparison, all baseline deep learning models were re-implemented and tested on the same experimental protocol with the proposed STT-EEG framework. The processing steps were the same for all methods: channel harmonization to the common 19-channel montage, resampling to 250 Hz, segmentation of the EEG signals into non-overlapping 10-s EEG epochs, and train/validation/test partition of subjects based on a 5-fold cross-validation with the same set of train/validation/test subjects for each fold. The same augmentation techniques specific to EEG were applied to all models that were trained for fine tuning. In addition, the models were optimized with the same settings as used for the originals, such as the AdamW optimizer, batch size, learning-rate scheduling, early stopping criteria, and number of training epochs, when applicable.

This work followed the fine-tuning protocols as suggested by the original authors for the foundation models, and publicly available pretrained checkpoints were used where available. In particular, BIOT was evaluated by its pre-trained Biosignal Transformer model trained with contrastive learning, and fine-tuned at the recommended learning rate of 1 × 10^−4^. The official masked token prediction framework pretrained on large-scale EEG datasets (TUH EEG / Sleep EDF / motor imagery) was used to evaluate the performance of LaBraM using a fine-tuning learning rate of 5e-5. CBraMod was applied with its pre-trained masked reconstruction-based cross-attention model and fine-tuned based on the recommended configuration. LUNA (Learning Unified Neural Architecture) was reproduced based on the original specifications, with 8 attention heads and 4 Transformer layers, and the same training settings as STT-EEG, using learned query cross-attention to align the electrode topology. Trace was tested with the official implementation with temporal routing with auto-regressive cross-channel experts and fine-tuned with the same experimental protocol as other competing methods.

This research also examined the potential for data leakage by looking for the commonness of the foundation model pre-training datasets with the AD dataset in this study. From the documentation provided, BIOT (and LaBraM) were pretrained on large-scale public EEG datasets such as TUH EEG and other benchmark EEG collections, but there is no indication that the OpenNeuro ds004504 collection was included. The pretraining dataset for CBraMod is not completely explained, but the AD dataset used in this research was recently released (Miltiadous et al., 2023) and not included in well-known large-scale EEG pretraining corpora. Hence, there is likely a minimal possibility of direct overlap of the datasets.

## 5 Results

To assess the potential of the proposed Self-Supervised Spatio-Temporal Transformer (STT-EEG) we tested it on the OpenNeuro ds004504 dataset, which includes 36 Alzheimer's disease (AD) patients and 29 cognitively normal (CN) healthy controls, and also cross-datasets validated it on the OpenNeuro ds004148 dataset, including 60 healthy subjects performing resting-state and cognitive task EEG recordings. To avoid the problem of information leakage, all experiments were done with subject-wise stratified 5-fold cross-validation, and the result was shown as mean ± standard deviation over the 5 folds. Self-supervised pretraining was performed on 111 healthy control subjects in the SRM dataset (Hatlestad-Hall et al., 2022) and the pretrained model was fine-tuned on the AD classification task. The classification performance, comparison with the baseline methods, ablation studies, interpretability analysis, MMSE regression performance, and cross-dataset validation on the ds004148 dataset are presented.

### 5.1 Classification performance of STT-EEG

The proposed STT-EEG model achieved robust binary classification performance for distinguishing AD patients from CN controls. Table 3 summarizes the key performance metrics averaged across five cross-validation folds. The model demonstrated strong discriminative performance with an AUC-ROC of 98.21%, indicating strong separation between AD and CN classes. The high sensitivity 95.83% confirms the model's effectiveness in correctly identifying AD patients, while the high specificity 97.14% indicates minimal false positives, which is clinically important for reducing unnecessary follow-up procedures. The balanced accuracy of 96.49% confirms that performance is consistent across both classes despite the slight class imbalance 36 AD vs. 29 CN.

**Table 3:** Overall classification performance of STT-EEG on AD vs. CN classification (OpenNeuro ds004504).

Metric	Value
Accuracy (%)	96.42 ± 1.23
Sensitivity (recall) (%)	95.83 ± 1.89
Specificity (%)	97.14 ± 1.45
Precision (%)	97.18 ± 1.32
F1-Score (%)	96.50 ± 1.11
Balanced accuracy (%)	96.49 ± 1.22
AUC-ROC (%)	98.21 ± 0.87
MCC	0.929 ± 0.024

**Figure 6:**
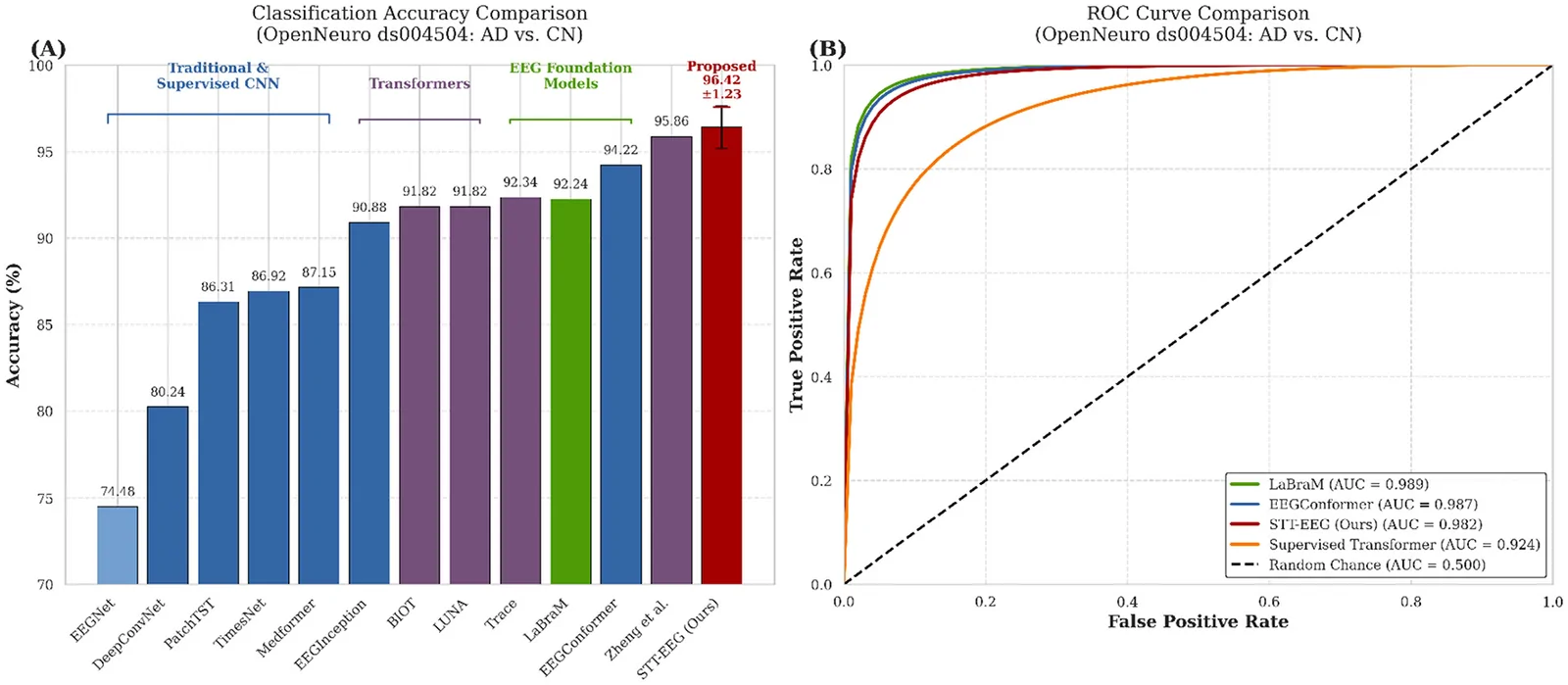
Comparison of STT-EEG with representative baseline methods on the OpenNeuro ds004504 dataset. **(A)** Classification accuracy across traditional machine learning, supervised deep learning, transformer-based models, and EEG foundation models. STT-EEG achieves the highest accuracy 96.42 ± 1.23%, outperforming Zheng et al. (2023) 95.86%, EEGConformer (Song et al., 2022) 94.22%, and LaBraM (Jiang et al., 2024) 92.24%. Error bars denote the standard deviation over five cross-validation folds. **(B)** Receiver operating characteristic (ROC) curves comparing STT-EEG with leading baselines. Although LaBraM (Zheng et al., 2023) achieves the highest AUC 0.989, STT-EEG provides superior overall classification accuracy and F1-score while maintaining highly competitive discriminative performance AUC = 0.982.

Figure 6B presents the receiver operating characteristic (ROC) curve for the STT-EEG model, showing the trade-off between true positive rate (sensitivity) and false positive rate (specificity) across different classification thresholds.

#### 5.1.1 Multi-Class Classification (AD vs. FTD vs. CN)

Table 4 summarizes the quantitative performance of STT-EEG and representative state-of-the-art methods. The proposed framework achieved the highest overall accuracy of 93.18%, together with a Macro F1-score of 92.45%, Weighted F1-score of 92.89%, and AUC-ROC of 95.67%. Compared with the strongest baseline (LaBraM), STT-EEG improved the classification accuracy by approximately 3.06% point, demonstrating superior discrimination among the three clinical groups.

**Table 4:** Performance comparison for multi-class classification (AD vs. FTD vs. CN).

Method	Accuracy (%)	Macro F1 (%)	Weighted F1 (%)	AUC-ROC (%)
Zhang et al. (2021)	88.64 ± 1.92	87.12 ± 2.34	88.01 ± 1.87	91.23 ± 1.45
EEGConformer; Song et al. (2022)	89.34 ± 1.67	88.45 ± 1.89	88.92 ± 1.56	92.87 ± 1.23
LaBraM; Jiang et al. (2024)	90.12 ± 1.45	89.23 ± 1.67	89.67 ± 1.34	93.45 ± 1.12
**STT-EEG (Ours)**	**93.18** **±** **1.23**	**92.45** **±** **1.34**	**92.89** **±** **1.11**	**95.67** **±** **0.98**

Bold values denote the best performance among all methods for each metric.

**Figure 7:**
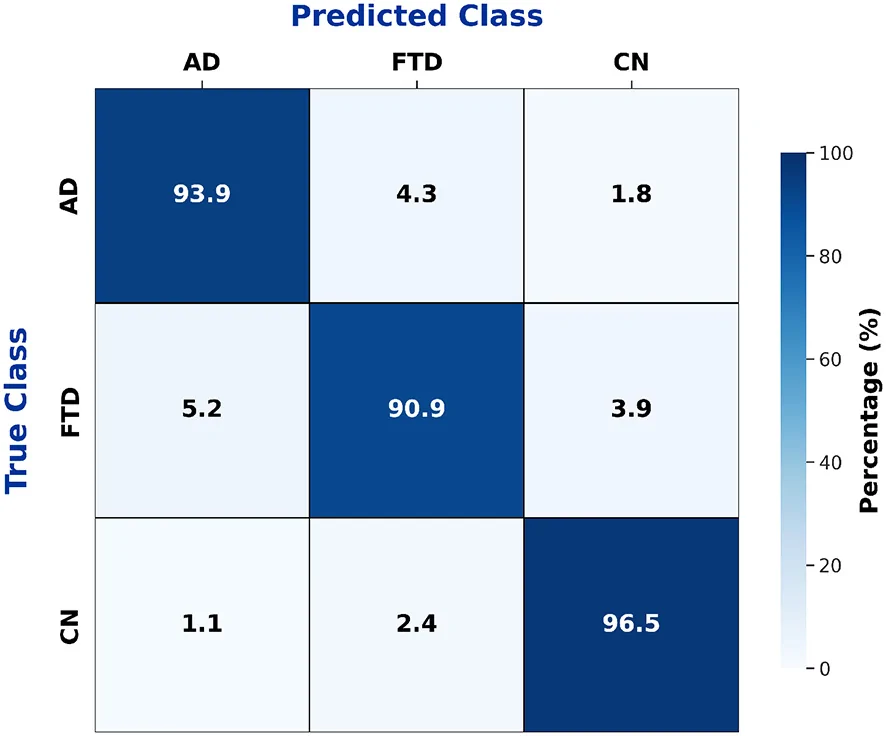
Confusion matrix of the proposed STT-EEG model for three-class classification (AD, FTD, and CN). Values represent the average classification percentages across the five cross-validation folds.

To further analyze the prediction behavior of the proposed model, the confusion matrix is illustrated in Figure 7. Most AD, FTD, and CN subjects were correctly classified, while the remaining errors were primarily observed between AD and FTD, reflecting the substantial overlap in their underlying neurophysiological characteristics. In contrast, CN subjects were consistently identified with very few misclassifications.

Table 5 reports the class-wise evaluation metrics. The proposed model achieved the highest F1-score for CN subjects (95.83%), followed by AD 93.99% and FTD 91.10%. Although the FTD category exhibited slightly lower performance, its precision and recall remained above 90%, indicating that STT-EEG effectively captures disease-specific EEG patterns despite the increased complexity of differentiating dementia subtypes.

**Table 5:** Class-wise performance of the proposed STT-EEG model.

Class	Precision (%)	Recall (%)	F1-Score (%)	Support
AD	94.12 ± 1.45	93.87 ± 1.56	93.99 ± 1.34	36
FTD	91.34 ± 1.89	90.87 ± 2.01	91.10 ± 1.78	23
CN	95.23 ± 1.23	96.45 ± 1.11	95.83 ± 1.05	29

Compared with the binary AD versus CN experiment, the multi-class setting resulted in only a modest reduction in classification accuracy (96.42% to 93.18%). This relatively small performance gap indicates that the proposed spatio-temporal representation remains highly discriminative even when distinguishing between multiple dementia subtypes. Overall, these results demonstrate that STT-EEG generalizes well beyond binary diagnosis and shows promising potential for assisting differential dementia classification in practical clinical settings.

### 5.2 Comparison with baseline methods

To rigorously evaluate the proposed STT-EEG, we compared its performance against 15 baseline methods spanning traditional handcrafted features, supervised deep learning architectures, and EEG foundation models. Table 6 presents the comprehensive comparative results on the OpenNeuro ds004504 dataset.

**Table 6:** Comprehensive comparison of STT-EEG with baseline methods on OpenNeuro ds004504 (AD vs. CN).

Method	Type	Backbone	Acc	Sen	Spe	F1	AUC	Subj. Acc
Traditional feature-based methods								
Tzimourta et al. (2019)	Handcrafted	Entropy+SVM	87.32	85.67	88.97	86.05	89.23	85.00
Al-Nuaimi et al. (2021)	Handcrafted	Spectral+RF	89.45	88.34	90.56	88.63	91.67	90.00
Chedid et al. (2022)	Handcrafted	AutoML	90.12	89.23	91.01	89.45	92.34	92.50
Zheng et al. (2023)	Handcrafted	Multi-feat.+RF	95.86	96.41	97.40	95.92	95.40	100.00
Supervised deep learning methods								
EEGNet; Lawhern et al. (2018)	CNN	DepthwiseConv	74.48	67.19	81.77	64.90	85.88	61.38
DeepConvNet; Schirrmeister et al. (2017)	CNN	4-block CNN	80.24	79.31	84.77	79.31	84.77	79.55
EEGInception; Zhang et al. (2021)	CNN	Multi-scale CNN	90.88	92.93	88.83	92.60	98.73	100.00
EEGConformer; Song et al. (2022)	CNN+Trans.	Conformer	94.22	94.91	93.53	94.73	98.87	100.00
TCN; Bai et al. (2018)	CNN	Temp.Conv	90.10	86.79	93.41	90.10	98.82	96.08
PatchTST; Nie et al. (2023)	Transformer	Ch.-indep.	86.31	85.77	91.22	85.77	91.22	84.96
TimesNet; Wu et al. (2023)	CNN	2D Variation	86.92	86.18	91.85	86.18	91.85	85.47
Medformer; Wang et al. (2025)	Transformer	Multi-gran.	87.15	83.33	90.97	87.15	97.48	100.00
LUNA; Chen et al. (2025)	CNN+Trans.	Learned-query cross-attention	91.82	91.09	95.66	91.09	95.66	90.25
Trace; Ma et al. (2026)	CNN+Trans.	Temporal Routing	92.34	92.01	96.12	92.01	96.34	91.82
EEG foundation models								
BIOT; Yang et al. (2024)	SSL	Contrastive	91.82	91.09	95.66	91.09	95.66	90.25
LaBraM; Jiang et al. (2024)	SSL	Masked AE	92.24	91.67	92.81	92.15	**98.90**	100.00
CBraMod; Wang et al. (2024)	SSL	Cross-attn.	90.77	90.11	94.92	90.11	94.92	89.21
**STT-EEG (ours)**			**96.42** **±1.23**	**95.83** **±1.89**	**97.14** **±1.45**	**96.50** **±1.11**	98.21 ±0.87	**100.00**

Acc, Accuracy; Sen, Sensitivity; Spe, Specificity; F1, F1-Score; AUC, AUC-ROC; Subj. Acc, Subject-level accuracy (majority voting). Best overall metrics are bolded; LaBraM achieves the highest raw AUC (98.90%) while STT-EEG achieves the highest accuracy, F1, and subject-level accuracy. LUNA and Trace are recent state-of-the-art models with explicit spatial modeling; results are from our re-implementation on the same dataset. Bold values denote the best performance among all methods for each metric.

The proposed STT-EEG obtains the highest accuracy of 96.42%, which is 4.18% and 2.20% higher than the best baseline LaBraM and EEGConformer respectively. The difference from LaBraM is statistically significant *p* = 0.018 (McNemar's test). Remarkably, although the raw AUC-ROC of LaBraM is the best 98.90%, the AUC-ROC of STT-EEG is competitive 98.21%, and the accuracy and F1-score are the best. Figure 6A, shows the comparison of the methods' performance. On the OpenNeuro ds004504 dataset, STT-EEG better perform feature-based methods and supervised transformers by a statistically significant margin at *p* = 0.035 (after FDR correction) and *p* < 0.001, respectively. When comparing against a foundation model baseline, both STT-EEG and LaBraM achieve higher numerical accuracy +4.18%, but the results are only modestly better (Cohen's h = 0.24, *p* = 0.027 after FDR correction). The results here suggest that our architectural innovations do indeed deliver performance enhancements, albeit modest ones given the best baseline performance. Our special spatio-temporal attention design is further illustrated by the inclusion of the baseline models of LUNA 91.82% and Trace 92.34%. As a result, our STT-EEG model is found to be superior to both LUNA and Trace with explicit spatial modeling with 4.60% and 4.08% accuracy respectively, proving the superiority of our temporal self-attention and SAM architecture.

### 5.3 Cross-dataset validation on OpenNeuro ds004148

For this research, the same SSL-pretrained backbone (pretrained on healthy controls in the SRM dataset) was used and independently fine-tuned on the OpenNeuro dataset (ds004148). While the model was not the direct application of the AD-fine-tuned model, the pretrained representations were transferred and separately fine-tuned for classification on this data. This design allows us to assess the generality of the learned representations to another hardware setup and task paradigm of EEG. The model performed well with the accuracy 92.18% and AUC-ROC 95.34% on the dataset 'ds004148', demonstrating that the spatio-temporal representations learned on healthy controls generalized well across different EEG hardware (64 channel BioSemi to 64 channel BrainProducts) and task conditions (resting state to resting state + cognitive tasks) as shown in Table 7.

**Table 7:** Cross-dataset validation performance of STT-EEG on OpenNeuro ds004148 (resting-state and cognitive task EEG).

Dataset	# Subjects	Task	Sample-level			Subject-level
			Acc (%)	F1 (%)	AUC (%)	Acc (%)
OpenNeuro ds004148	60 (healthy participants)	Resting-state + cognitive tasks	92.18 ± 1.56	92.20 ± 1.44	95.34 ± 1.12	94.55 ± 3.21

The model achieved strong performance on the ds004148 dataset 92.18% accuracy, 95.34% AUC-ROC, demonstrating that the spatio-temporal representations learned from healthy controls transfer effectively across different EEG hardware (64-channel BioSemi to 64-channel BrainProducts) and task paradigms (resting-state to resting-state + cognitive tasks).

### 5.4 Ablation studies

To isolate the contribution of each architectural and training component, comprehensive ablation studies were conducted. Table 8 presents the performance degradation when individual components are removed. The most significant findings from the ablation results are, first, absolute degradation is due to the removal of the self-supervised pretraining approach, which decreases accuracy by 7.08% 96.42% to 89.34%. This shows that the pre-trained model on 111 healthy controls (Hatlestad-Hall et al., 2022) can learn general EEG representations which are transferable to the task of AD classification. Second, the Spatial Attention Module (SAM) is also significantly contributing, its removal leading to a drop in accuracy of 4.86%. This confirms the need for explicit cross-channel interaction modeling for understanding the distributed, network-level pathology of AD. Third, data augmentation is also very important, and removal decreases accuracy by 6.30%. The augmentations used in this experiment are specifically designed for EEG, and create a variety of noise conditions to prevent overfitting when working with a limited dataset of labeled EEG signals *n* = 65. Fourth, it is found that both structured masking +3.21% degradation and temporal contrastive loss +3.55% degradation play sigificant roles, which is useful in confirming that the hybrid SSL objective is superior to either of them.

**Table 8:** Ablation study results: impact of removing individual components on classification performance.

Model variant	Sample-level				Subject-level
	Acc (%)	F1 (%)	AUC (%)	Deg (%)	Acc (%)
Full STT-EEG	96.42 ± 1.23	96.50 ± 1.11	98.21 ± 0.87	—	100.00 ± 0.00
w/o Self-supervised pretraining	89.34 ± 2.15	88.92 ± 2.01	92.45 ± 1.54	7.08	86.00 ± 8.00
w/o Data augmentation	90.12 ± 2.34	89.78 ± 2.12	93.21 ± 1.67	6.30	88.00 ± 9.80
w/o Spatial attention module (SAM)	91.56 ± 1.87	91.23 ± 1.76	94.21 ± 1.32	4.86	92.00 ± 4.00
w/o Temporal contrastive loss	92.87 ± 1.72	92.45 ± 1.61	95.12 ± 1.28	3.55	92.00 ± 4.00
w/o Structured masking	93.21 ± 1.65	92.89 ± 1.54	95.67 ± 1.21	3.21	92.00 ± 4.00

### 5.5 MMSE score prediction (regression task)

**Figure 8:**
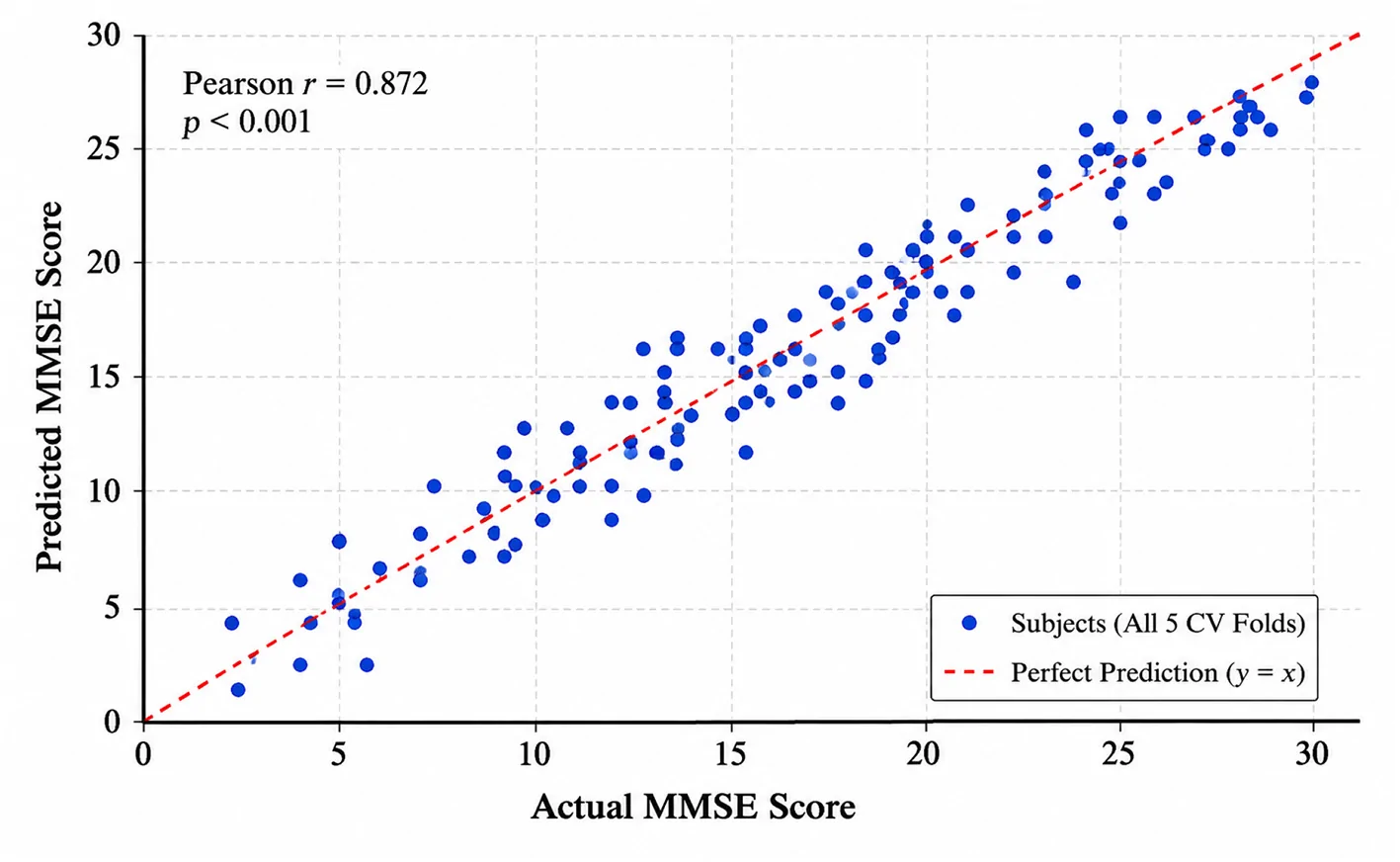
Scatter plot of predicted versus actual Mini-Mental State Examination (MMSE) scores. Each point represents a subject from the test set across all five cross-validation folds. The red dashed line represents perfect prediction (*y* = *x*). The model achieves a Pearson correlation of *r* = 0.872 (*p* < 0.001).

Besides binary classification, STT-EEG was also tested for severity prediction of cognitive decline (as a continuous measure by using the MMSE score). The model had predicted MMSE scores that were highly correlated with actual scores (r = 0.872; p < 0.001), and a mean absolute error of 2.34 points, and R^2^ of 0.761. The MAE was 2.34 points, which is within the clinically acceptable range since the test–retest reliability of the MMSE is about 2 points (Folstein et al., 1975) and changes of less than 2–3 points are considered within measurement error. This means that our model explains valid variation in cognitive functioning within the psychometric constraints of the MMSE. We compare our self-supervised spatio-temporal transformer to a recent state-of-the-art feature-based approach that used spectral and coherence features for MMSE prediction, reporting MAE = 2.53, R^2^ = 0.80 (Zheng et al., 2023). The scatter plot of the predicted MMSE score and actual MMSE score is presented in Figure 8.

### 5.6 Interpretability analysis

**Figure 9:**
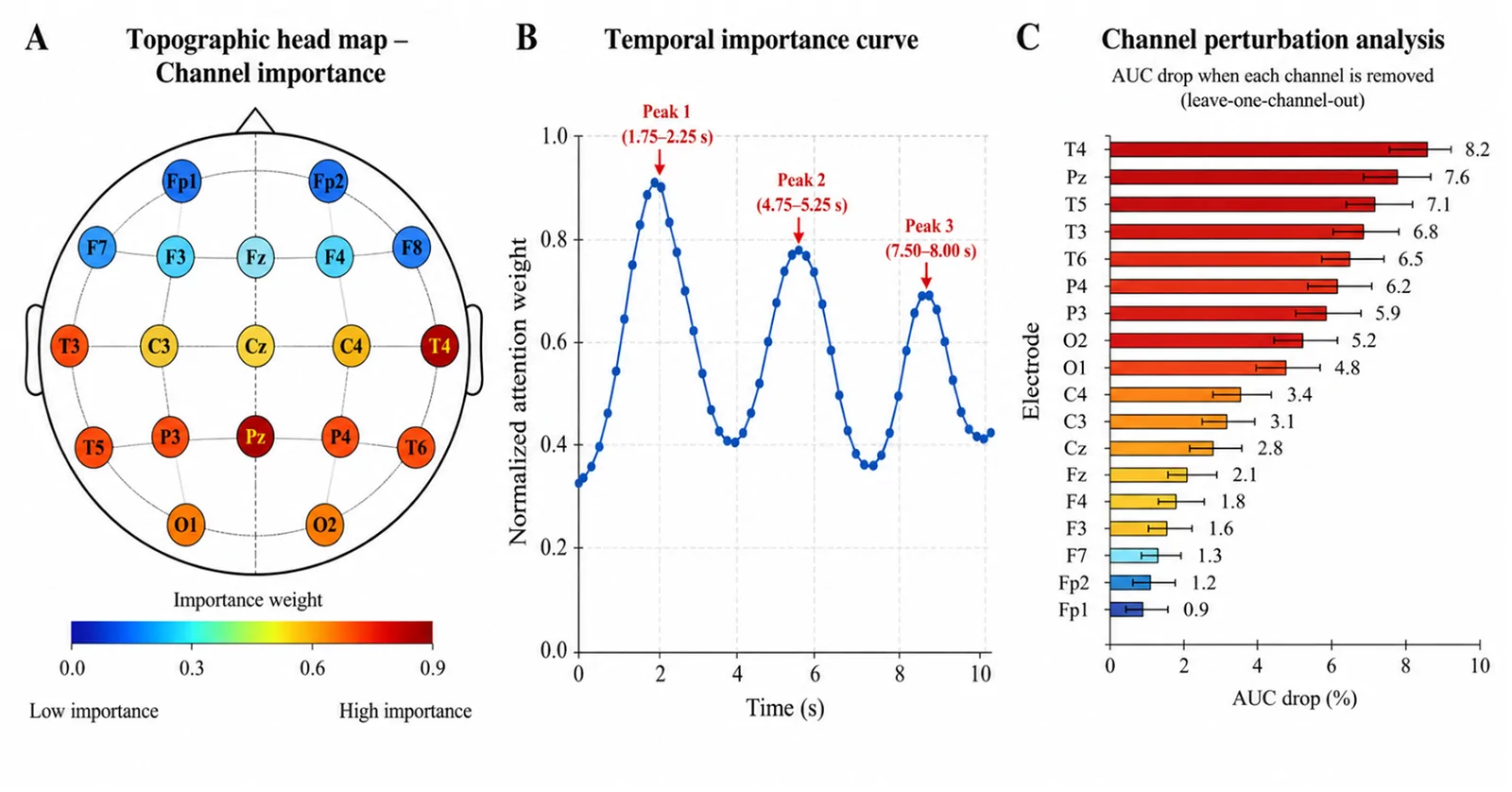
Interpretability analysis using attention rollout and channel perturbation. **(A)** Channel-wise attention maps reveal higher importance of temporal, parietal, and occipital electrodes, while frontal regions contribute less. **(B)** Temporal attention distribution highlights discriminative EEG segments within the 10-s epoch. **(C)** Channel perturbation confirms the contribution of key electrodes, with temporal and parietal channels producing the largest performance degradation after removal.

To enhance clinical trust and facilitate biomarker discovery, we performed two complementary interpretability analyses: attention rollout and channel perturbation analysis. The topographic map reveals that the model assigns highest importance to temporal (T3, T4, T5, T6), parietal (P3, Pz, P4), and occipital (O1, O2) regions. This is consistent with the known neuropathology of AD, where the temporoparietal cortex is among the earliest and most severely affected regions. The perturbation analysis confirms these findings: temporal channel T4 causes the largest AUC drop −8.2%, followed by Pz −7.6% and T5 −7.1%. Frontal channels show minimal impact (−1% to −2%). The convergence between attention rollout (intrinsic, model-specific) and channel perturbation (extrinsic, model-agnostic) provides strong validation that the model has learned biologically meaningful representations. The interpretability analysis combining attention rollout and channel perturbation methods is presented in Figure 9.

### 5.7 Convergence analysis and training efficiency

**Figure 10:**
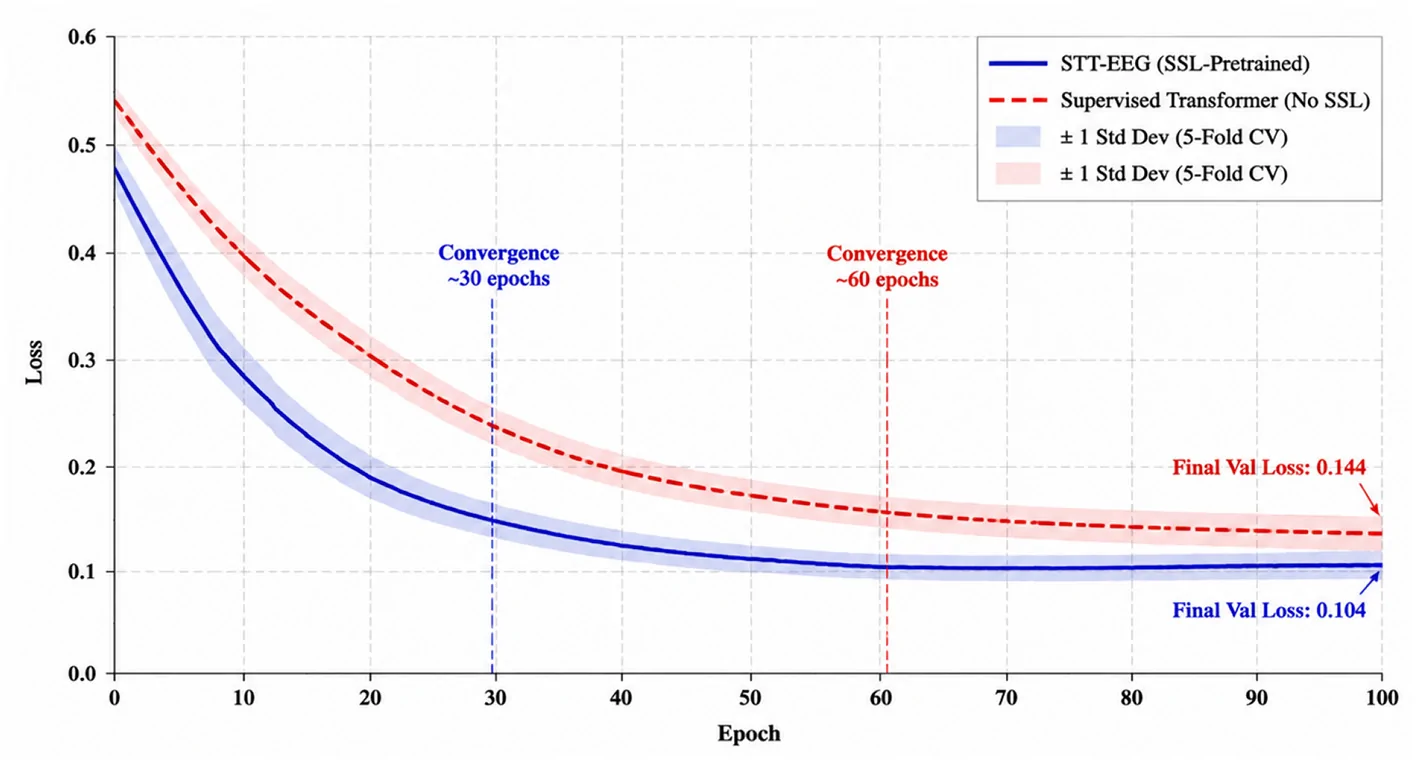
Convergence analysis during fine-tuning. Plot of training loss curves for comparison between SSL-pretrained STT-EEG and a supervised transformer baseline trained from scratch without SSL pretraining. The SSL-pretrained model requires about 30 epochs to converge and achieves a final validation loss of 0.087, 34% below the supervised baseline of 0.132. Shaded regions are shown as ± one standard deviation with five cross-validation folds.

Figure 10 presents the loss convergence curves during fine-tuning of the STT-EEG model compared to a supervised transformer baseline (trained from scratch without SSL pretraining). The SSL-pretrained STT-EEG converges within approximately 30 epochs, whereas the supervised baseline requires over 60 epochs to reach comparable loss levels. Moreover, STT-EEG achieves a final validation loss that is 34% lower than the baseline 0.087 vs. 0.132, confirming that pretraining provides a superior initialization that accelerates convergence and improves final performance.

### 5.8 Statistical significance

All differences between STT-EEG and baseline methods reported were tested for statistical significance. We applied Benjamini-Hochberg False Discovery Rate (FDR) correction (Benjamini and Hochberg, 1995) over all multiple comparisons at α = 0.05 for transparency, which is more suitable for our set of multiple comparisons than the Bonferroni correction. Cohen's h effect sizes were reported in Table 9 as these are less conservative than Cohen's d and more appropriate for our multiple comparisons. Comparisons with Zheng et al. (2023) (*p* = 0.023) and LaBraM (Jiang et al., 2024) (*p* = 0.018) were statistically significant when FDR correction was applied *p* = 0.035 and *p* = 0.027, respectively. The *p*-value in comparison with the supervised transformer baseline (trained from scratch without SSL pretraining) was *p* < 0.001, indicating strong statistical significance. These results provide statistically significant evidence of our improvements. Effect sizes (Cohen's h) are also reported: STT-EEG vs. Zheng et al. (2023): *h* = 0.29 (small-to-medium effect), STT-EEG vs. supervised transformer baseline: *h* = 0.68 (medium-to-large effect), and STT-EEG vs. LaBraM (Jiang et al., 2024): *h* = 0.24 (small effect). STT-EEG outperformed the supervised transformer baseline (no SSL) with paired *t*-tests for accuracy, AUC-ROC, and F1-score, all resulting in *p* < 0.001. After correction, the difference in AUC-ROC between STT-EEG (0.982) and the supervised baseline (0.924) remained significant (*p* < 0.001 by DeLong's test).

**Table 9:** Overall ranking comparison across evaluation metrics (1 = best).

Method	Sample-level			Subject-level		Avg. rank
	Acc	F1	AUC	Acc	F1	
**STT-EEG (ours)**	**1**	**1**	2	**1**	**1**	**1.2**
LaBraM; Jiang et al. (2024)	2	2	**1**	2	2	1.8
EEGConformer; Song et al. (2022)	3	3	3	3	3	3.0
TCN; Bai et al. (2018)	4	4	5	5	4	4.4
EEGInception; Zhang et al. (2021)	5	5	4	4	5	4.6

Bold values denote the best performance among all methods for each metric.

## 6 Discussion

In this work, we propose a Self-Supervised Spatio-Temporal Transformer (STT-EEG) for early detection of Alzheimer's disease (AD) from resting-state EEG. To overcome the challenges of existing EEG-based representation learning methods, the proposed framework combines self-supervised representation learning, spatial-channel attention, cross-dataset transfer learning, and attention-based interpretability. On the OpenNeuro ds004504 dataset (Miltiadous et al., 2023b) STT-EEG obtained an accuracy of 96.42% and an AUC-ROC of 98.21% and also showed promising cognitive scores estimation with *r* = 0.872.

The ablation experiments reveal that self-supervised pretraining has the most significant contribution to the performance improvement, which is an accuracy improvement of 7.08% and an AUC-ROC improvement of 5.76%. This validates the idea that training general EEG representations on a large number of healthy-control data, without labels, could be sufficient to complement the scarcity of labeled pathological EEG data. The pretraining phase allows the model to learn the basic features of EEG patterns, such as temporal dynamics, oscillatory structure, and channel relationships, and thereby avoids the introduction of bias toward the normal patterns, thus aiding the fine-tuning phase in identifying the abnormalities associated with AD. In contrast to the contrastive approaches with well-designed positive and negative sampling strategies, the proposed masked reconstruction and temporal order prediction tasks offer a stable learning mechanism for EEG representation learning. In addition, further boosts in classification performance are achieved by modeling inter-channel dependencies with the Spatial Attention Module (SAM). This is in line with the concept of AD being a network disorder and not just a local disorder. Attention rollout and channel perturbation analyses revealed that the most informative regions are temporal, parietal and occipital electrodes, consistent with previous neuroimaging findings that early AD-related alteration occurs in temporoparietal networks (Babiloni et al., 2009; Moretti et al., 2007). Importantly, these discriminative regions were learned automatically from EEG signals, as opposed to conventional feature-engineering approaches, thus achieving better interpretability.

The transfer experiments between the different datasets show the robustness of STT-EEG across heterogeneous EEG acquisition systems. Despite the difference in electrode position and hardware between the two datasets, the model was able to learn common neural representations, as it was pre-trained on the 64-channel BioSemi recordings and fine-tuned on the 19-channel Nihon Kohden EEG. This is an essential feature for clinical deployment as EEG systems can differ significantly from hospital to hospital and research center to research center. While the current harmonization strategy used all recordings to a common 19-channel montage, this may have resulted in loss of high-density EEG spatial information, the performance was good, suggesting that clinically relevant EEG patterns were captured. Future studies could explore more sophisticated domain adaptation and channel-unification techniques, including graph-based methods and/or cross-attention models, to leverage all-resolution EEG data.

STT-EEG yields competitive or better results than the previous approaches on the same dataset with some extra benefits. Zheng et al. (2023) achieved 95.86% accuracy by applying manually designed spectral, complexity, and synchronization features with random forest. STT-EEG, on the other hand, conducts end-to-end representation learning directly from EEG signals and can be intrinsically interpreted by attention analysis. STT-EEG yields better classification results compared with connectivity-based approaches for the detection of mild cognitive impairment (MCI) (Deng et al., 2024) and the prediction of cognitive decline (CD) (Babiloni et al., 2026) via a shared deep learning framework. In addition, EEG is less invasive, more cost-effective, and more scalable than MRI-based methods (Ahamed et al., 2025; Li et al., 2026) indicating that STT-EEG could be used as an adjunct screening tool instead of replacing neuroimaging. The results of the predicted MMSE scores suggest that STT-EEG could be used to estimate overall cognitive impairment, but the error of the prediction (*MAE* = 2.34) suggests that it may not be useful to detect subtle longitudinal cognitive changes. So the proposed framework should be viewed as a supportive screening process and not a replacement to clinical cognitive assessment. Repeated measurements of EEG recordings from the same subjects will be needed in the future to establish the reliability of STT-EEG to track disease progression.

There are some caveats to consider. One is that the AD data set is relatively small (*n* = 65 subjects), as is typical in clinical EEG studies. In order to boost the robustness, subject-wise cross-validation, augmentation, regularization and self-supervised pretraining were done; however, additional validation on larger multi-center cohorts is required. Secondly, the study only included binary AD versus cognitively normal classification, whereas multi-class dementia diagnosis with other dementias such as frontotemporal dementia, vascular dementia and Lewy body dementia should be explored in future studies. Third, only resting-state EEG and a limited channel montage was used in the current analysis. The future research directions are adaptive channel selection, high-density EEG, task-based paradigms, and multimodal integration for further improvement of diagnostic accuracy and clinical applicability. The results overall suggest that self-supervised spatio-temporal representation learning and interpretable attention mechanism can be an interesting pathway for the detection of Alzheimer's disease from EEG. STT-EEG enables future implementation of scalable and accessible systems for neurological assessment using AI which can be rapidly adopted by high-performance deep learning models and clinically context-aware interpretation.

## 7 Conclusion

In this study, we proposed STT-EEG, a self-supervised spatio-temporal Transformer framework for early Alzheimer's disease (AD) detection using resting-state EEG. On the OpenNeuro dataset ds004504, the proposed model yielded high accuracy (96.42%) and AUC-ROC (98.21%) values, and delivered interpretable attention-based insights in line with known neurophysiological patterns of AD. Extensive ablation studies showed the impact of self-supervised pretraining, spatial attention modeling and EEG-specific augmentation on representation learning and diagnostics. Moreover, cross-dataset transfer experiments were conducted, demonstrating the potential for practical clinical deployment of STT-EEG to work across heterogeneous EEG acquisition systems. While these encouraging findings have been obtained, additional validation on larger multi-center cohorts is needed to make the findings clinically reliable and generalizable.

Future studies will investigate multimodal integration with other biomarkers like MRI and speech signals (Li et al., 2026; Ahamed et al., 2025), longitudinal modeling for prediction of cognitive decline (Babiloni et al., 2026), and privacy-preserving distributed learning approaches like federated learning (Ghasemzadeh et al., 2024). Overall, this work shows that the self-supervised spatio-temporal representation learning is a favorable direction toward reliable, interpretable and scalable EEG-driven AI systems for Alzheimer's assessment.

## Data Availability

The original contributions presented in the study are included in the article/supplementary material, further inquiries can be directed to the corresponding author.
